# Applications of Nanotechnology in Athletic and Exercise Performance: A Scoping Review

**DOI:** 10.3390/jfmk11040416

**Published:** 2026-10-13

**Authors:** Rocco De Vitis, Luca Lombardi, Matteo Guzzini, Arturo Militerno, Giuseppe Taccardo, Marco Passiatore

**Affiliations:** 1 Department of Orthopaedic and Geriatric Sciences, Fondazione Policlinico Universitario Agostino Gemelli IRCCS, 00168 Rome, Italy; arturo.militerno@policlinicogemelli.it (A.M.); giuseppe.taccardo@guest.policlinicogemelli.it (G.T.); 2 Independent Researcher, Fisioterm Rehabilitation Center, 00189 Rome, Italy; lucalombardi@fisioterm.com; 3 Department of Orthopaedic, Uni-Camillus, International Medical University, 00131 Rome, Italy; matteo.guzzini@unicamillus.org; 4 Department of Bone and Joint Surgery, University of Brescia, 25123 Brescia, Italy; marco.passiatore@unibs.it

**Keywords:** nanotechnology, athletic performance, carbon nanotubes, nanoparticles, biosensing techniques, quantum dots, photobiomodulation therapy, wearable electronic devices, exercise, oxidative stress

## Abstract

Nanotechnology, the science of materials engineered at the 1–100 nanometer (nm) scale, is increasingly applied across domains relevant to elite sport, including equipment engineering, wearable biosensing, nanoparticle-based supplementation, photobiomodulation (PBM) nanotechnology, and quantum dot (QD)-enabled diagnostics; however, the aggregate impact of these applications on measurable athletic performance outcomes remains poorly synthesized. This systematic scoping review, conducted and reported in accordance with PRISMA-ScR, aimed to map and characterize available evidence on nanotechnological applications relevant to athletic and exercise performance across six domains: carbon nanotube (CNT)-reinforced equipment, nano-antioxidant supplementation, nanoparticle-based nutrient delivery, wearable nanosensors, PBM nanotechnology, and QD-based biosensing. A systematic search was performed in PubMed/MEDLINE, Scopus, and Web of Science (inception to 15 June 2026). Eligibility was assessed through four evidence strata: Stratum 1, RCTs and controlled studies in human participants of any training status (direct controlled human evidence; stratum classification determined by study design; population training status recorded as a separate determinant of interpretive generalizability within the stratum); Stratum 2, observational and experimental studies in healthy humans; Stratum 3, validated animal exercise models (restricted a priori to the nano-supplementation domain); and Stratum 4, subdivided into Stratum 4a (systematic reviews, meta-analyses, narrative/comprehensive reviews) and Stratum 4b (materials science studies). Quality appraisal was performed using study-design-specific tools (RoB 2, NOS, SYRCLE, AMSTAR-2, structured narrative frameworks). No formal GRADE certainty-of-evidence assessment was performed. A total of 28 records met inclusion criteria: 12 primary studies across Strata 1–3 and 16 secondary or contextual records in Stratum 4. Direct controlled human evidence (Strata 1–2) was identified for CNT-based insoles, Taopatch^®^ PBM devices, and CNT motion sensors; animal model evidence (Stratum 3) was consistent for nano-antioxidant strategies; and Stratum 4 provided contextual technical and mechanistic framing. No Stratum 1 study applied QDs to elite athletes in vivo. Nanotechnology demonstrates theoretical and emerging translational potential across several domains relevant to athletic and exercise performance; however, direct evidence of meaningful performance enhancement in trained or elite athletes remains very limited. Adequately powered double-blind RCTs in elite athletes, independent nanomaterial characterization, and prospective protocol registration are required before clinical recommendations can be formulated.

## 1. Introduction

Nanotechnology, broadly defined as the manipulation of matter at dimensions between 1 and 100 nm, has undergone a transformative expansion in biomedical and materials science over the past two decades [1]. The unique physicochemical properties conferred at the nanoscale, including exceptional strength-to-weight ratios, high surface area-to-volume ratios, quantum confinement effects, and tunable optical or electrical characteristics, have positioned nanomaterials as a pivotal frontier in translational research with direct implications for human physical performance [2].

In the context of elite sport, athletic performance is determined by a complex interplay of physiological, biomechanical, nutritional, and neurophysiological variables. In elite sport, even small biomechanical, physiological, or technological improvements may contribute to meaningful performance advantages. In the context of nanotechnology, such potential “marginal gains” are most plausibly reflected in equipment engineering, wearable monitoring, and nano-enabled recovery strategies [2,3]. Nanotechnological innovations offer potential contributions across several of these dimensions:

- Equipment engineering: CNTs and graphene-reinforced composites have been incorporated into racquets, bicycle frames, running shoes, and protective gear, yielding materials that are simultaneously lighter and mechanically superior to conventional alternatives [4,5].
- Nano-enabled supplementation: Nanoparticle encapsulation of bioactive compounds, including resveratrol, coenzyme Q10 (CoQ10), and antioxidant molecules, has been investigated as a strategy to overcome poor bioavailability and achieve targeted delivery to sites of exercise-induced oxidative stress [6,7,8].
- Wearable nanosensors: Flexible CNT- and nanomaterial-based strain sensors, and electrochemical biosensors enabling real-time sweat metabolite monitoring (lactate, glucose, electrolytes), represent a paradigm shift in performance monitoring and athlete physiology [9,10].
- Photobiomodulation nanotechnology: Devices such as Taopatch^®^ (Tao Technologies Srls, Cittadella, Italy), CE-marked as a Class I medical device in the European Union under Regulation 2017/745, utilize photoselective nanomaterials that, according to the manufacturer, are activated by body-heat-generated infrared radiation and claimed to modulate cutaneous photoreceptors and proprioceptive afferents through quantum confinement-analogous optical principles. This mechanistic characterization has not been independently validated in peer-reviewed nanotechnology or spectroscopic literature, and the CE Class I marking reflects general safety and performance requirements, not a regulatory demonstration of clinical efficacy. These devices have been investigated for effects on postural control and balance in healthy and clinical populations [11,12], although the evidence base remains preliminary and methodologically limited.
- Quantum dots (QDs): Semiconductor nanocrystals with tunable fluorescent properties have extensive biomedical imaging and biosensing applications, with theoretical relevance to real-time physiological monitoring in sport science [13,14].

Despite growing interest, to the authors’ knowledge, no prior systematic scoping review has comprehensively mapped and characterized evidence across all nanotechnological sub-domains specifically in the context of athletic and exercise performance.

The heterogeneity of the available evidence base, spanning direct athlete RCTs, translational human studies, animal exercise models, materials science investigations, and contextual systematic reviews, means that a standard systematic review with homogeneous PICOS eligibility criteria is not the appropriate methodological framework for an initial evidence synthesis in this emerging domain.

The present work is therefore designed and reported as a systematic scoping review, following the PRISMA Extension for Scoping Reviews (PRISMA-ScR) [15,16] as the primary reporting framework. For clarity, the following operational definitions apply throughout this review: “nanotechnology” refers to the design, characterization, production, and application of structures, devices, and systems in the 1–100 nm range; “nanomaterial” refers to a material with at least one external dimension or internal structure at the nanoscale; “nanocomposite” refers to a multi-component material in which at least one component has nanoscale dimensions and contributes to the bulk properties; and “nano-enabled device” refers to a device whose function depends on, or is substantially enhanced by, nanoscale components, even if the device as a whole is macroscopic. Some included sports-equipment applications involve hierarchical composites in which nanoscale constituents contribute to bulk properties; these are classified as nanotechnological applications and distinguished from purely macroscopic carbon-fiber products. Additionally, operational definitions for participant classifications are provided in Section 2.2.

This design explicitly accommodates the inclusion of heterogeneous evidence strata, including primary studies, reviews, and indirect translational evidence, enabling the mapping of the full scope of available knowledge while maintaining methodological transparency. It should be noted that, unlike the standard recommended by PRISMA 2020 Item 24 [15], this review was not prospectively registered on PROSPERO; this limitation is explicitly acknowledged and discussed in Section 4.5.

Because several sports-equipment applications involve hierarchical composites rather than isolated free nanomaterials, the term “nanotechnological application” was used to include materials in which nanoscale constituents, coatings, fillers, or nanoscale engineering principles contributed to the final device or material properties. Purely macroscopic carbon-fiber products without nanoscale components were included only when the source explicitly described nanoscale reinforcement or nano-engineered design features.

## 2. Methods

### 2.1. Protocol, Registration, and Reporting Standards

This systematic scoping review was designed and reported in accordance with the PRISMA-ScR checklist [16] as the primary reporting standard. Specific items from the PRISMA 2020 statement [15] are referenced only where directly applicable (e.g., reporting of the systematic search strategy, risk of bias assessment). Methodological requirements specific to systematic reviews in the PRISMA 2020 framework that are not applicable to a scoping review design, such as GRADE certainty-of-evidence assessment, were not applied; this is explicitly acknowledged throughout. The PRISMA-ScR checklist with indication of corresponding manuscript sections is provided in Supplementary Material Section S3.

The review protocol was not prospectively registered on PROSPERO (https://www.crd.york.ac.uk/prospero: URL accessed on 27 August 2026) prior to commencement of the search. This constitutes a methodological limitation of the present work: without prospective registration, the possibility of post hoc modifications to the search strategy, eligibility criteria, or outcome selection, which may introduce selective outcome reporting bias, cannot be excluded. This limitation is discussed further in Section 4.5. Prospective protocol registration on an appropriate public platform, such as OSF or PROSPERO where eligible, is strongly recommended for future updates. No amendments to the search strategy or eligibility criteria were made after data collection commenced; however, since no pre-registered protocol exists, this statement cannot be externally verified, and readers should interpret the findings with proportionate caution.

### 2.2. Eligibility Criteria

Given the heterogeneous and emerging nature of the evidence base, a Population–Concept–Context (PCC) framework [16] was adopted in preference to a standard PICOS approach, with explicit stratification of included studies into four evidence strata. This stratified framework directly addresses the breadth of available evidence types, from direct elite athlete RCTs to materials science characterization studies, in a methodologically transparent manner appropriate for a scoping review design.

#### PCC Framework

Population (P): Human participants of any age, sex, or training status, including healthy volunteers, recreational athletes, trained athletes, and elite athletes, enrolled in a controlled study design (Stratum 1, direct controlled human evidence); stratum classification is determined by study design, not by participant training status; the training status of enrolled participants (elite athlete, trained athlete, or healthy/non-elite subject) is recorded as a separately extracted characteristic that determines the directness and generalizability of Stratum 1 findings within the synthesis; studies enrolling healthy or non-elite participants that satisfy the controlled-design requirement are classified at the lower boundary of Stratum 1 and described as proof-of-concept controlled human evidence throughout the manuscript. Human subjects of any training status enrolled in non-controlled (observational or quasi-experimental) study designs without a concurrent control group (Stratum 2, indirect human evidence); validated animal exercise models (Stratum 3, translational evidence, restricted a priori to the nano-supplementation sub-domain given the absence of equivalent controlled human trial data—see justification below); and study populations within systematic reviews, narrative reviews, or materials science studies (Stratum 4, contextual framework evidence). For the purposes of this review, “elite athlete” is operationally defined as an individual nationally or internationally competitive in their primary sport discipline; “trained athlete” as an individual engaged in regular structured training with a sport-specific performance goal; “healthy subjects” as individuals without relevant medical conditions and not formally classified as trained or elite athletes. These definitions are applied consistently throughout and are acknowledged as a source of population heterogeneity.

Justification for restriction of animal studies to the nano-supplementation stratum (a priori decision): This restriction was established during protocol development, prior to the commencement of database searching. The rationale is as follows: in the nano-supplementation domain, the absence of any controlled human trial data means that translational animal evidence is the only available primary evidence source; the treadmill exhaustion models employed in this domain have established construct validity for exercise physiology research. In all other included domains (CNT-based equipment, wearable nanosensors, PBM nanotechnology, QD-based biosensing), the primary evidence base derives from human subjects, materials science characterization, or engineering validation studies, for which animal models do not represent a necessary or methodologically appropriate evidence tier. While the absence of prospective registration prevents external verification of this decision, the authors confirm that no modification to this criterion was made after database searching commenced.

For Stratum 4 materials science studies, the eligibility criterion of “at least one quantitative outcome relevant to athletic performance” was operationalized to include biomechanical performance outcomes (e.g., stiffness-to-weight ratio, wear resistance, gauge factor under high-strain conditions) that represent direct technical prerequisites for athletic performance applications, even when direct measurement of performance in athletes was not performed. The distinction between “technology-enabling outcomes” (demonstrating technical feasibility for eventual athletic application) and “direct athletic performance outcomes” (demonstrating improvement in a measured athlete parameter) is maintained throughout the synthesis; technology-enabling outcomes are classified exclusively as Stratum 4 contextual evidence.

Concept (C): Any nanotechnological application relevant to athletic or exercise performance, including but not limited to, CNT-based sports equipment and composites; nano-encapsulated nutritional supplements and antioxidants; nanosensor-based wearable monitoring systems; PBM nanotechnology devices (including Taopatch^®^); and QD-based biosensing systems.

Context (Co): Athletic performance, exercise physiology, sports biomechanics, physical rehabilitation with direct translational relevance to athletic populations, or materials science of sports equipment.

The exclusion of non-English language publications was applied at the full-text screening stage. This exclusion constitutes a source of language bias, particularly given the significant volume of nanotechnology research published in Chinese-language journals; this limitation is addressed in Section 4.5.

Stratified eligibility criteria are presented in Table 1 and eligibility exclusion criteria applied across all strata in Table 2.

Stratum 3 domain restriction: The a priori restriction of Stratum 3 to the nano-supplementation/nano-antioxidant domain was established during protocol development prior to search commencement. Animal exercise models evaluating nano-composite equipment or wearable nanomaterial devices were not identified in the search and would not have satisfied the Stratum 3 eligibility criteria as defined, as no validated animal performance model for these categories exists in the current literature. This restriction is consistent with the established use of rodent exhaustion models as the primary preclinical evidence base for nutritional and pharmacological exercise interventions, and with the absence of a direct translational pathway from animal models to CNT-composite equipment performance.

Stratum 4b PoC human component: Where a Stratum 4b materials science study includes a proof-of-concept human demonstration component, any data from the human component are extracted and reported descriptively but are not reclassified as Stratum 1 or Stratum 2 evidence. Reclassification would require a formal clinical sample size, defined eligibility criteria, defined primary outcome, and appropriate comparator as specified under Strata 1–2.

Stratum 4a PBM contextual framework evidence: Five true systematic reviews and meta-analyses of photobiomodulation therapy (PBMT/LLLT/LED) in human exercise populations (Studies 13–17) are included as Stratum 4a evidence. These reviews evaluate conventional characterised PBMT devices (with defined wavelength, irradiance, and energy dose parameters) rather than nanotechnological devices specifically. Their inclusion is explicitly justified in Section 2.2 of the main manuscript: they provide the dosimetric and clinical efficacy framework against which the Taopatch^®^ nanotechnology claims are evaluated and contextualised. They are labelled throughout the manuscript as “contextual PBM framework evidence” and are explicitly distinguished from primary nanotechnology-specific evidence. The critical translational limitation, that Taopatch^®^ emission spectrum and irradiance have not been independently characterised and therefore cannot be directly compared to the dosimetric parameters evaluated in these reviews, is stated at each point of citation.

Evidence hierarchy and interpretive weighting: The stratum classification is used for descriptive synthesis and evidence mapping, consistent with scoping review methodology. It is not used to formally grade evidence quality for clinical recommendation purposes, for which GRADE methodology would be required. Strata 1 and 2 carry the greatest direct interpretive weight for conclusions regarding human performance effects; Stratum 3 is interpreted as mechanistic and preliminary preclinical evidence only; Stratum 4a and 4b are interpreted as contextual, technical feasibility, and mechanistic framework evidence. No cross-stratum quantitative comparison of quality scores is made.

Inclusion criteria: Studies reporting at least one quantitative outcome relevant to athletic performance (primary: VO_2_max, power output, sprint time, jump height, time to exhaustion, endurance, or strength) or physiological biomarkers of direct exercise relevance (secondary: oxidative stress markers, blood lactate, muscle damage indices, postural control parameters, biomechanical outcomes), and involving at least one nanotechnological application as defined under the Concept dimension, were eligible for inclusion across all four strata.

Exclusion criteria: In vitro-only investigations without any in vivo or biomechanical outcome; case reports (n = 1) without quantitative performance outcomes; conference abstracts without full peer-reviewed text availability; non-peer-reviewed sources; and studies published in languages other than English. Animal studies were restricted to the nano-antioxidant sub-domain given the absence of equivalent controlled human trial data; animal studies from other domains were excluded. Non-sport biomedical studies without any direct or indirect translational relevance to exercise performance were excluded.

### 2.3. Search Strategy

Systematic searches were conducted independently in three electronic databases: PubMed/MEDLINE (inception to 15 June 2026), Scopus (inception to 15 June 2026), and Web of Science, Core Collection (inception to 15 June 2026). These databases were selected for the following reasons: PubMed/MEDLINE provides the most comprehensive coverage of biomedical, exercise physiology, and sports medicine literature indexed with MeSH controlled vocabulary; Scopus provides broad multidisciplinary coverage, including materials science, engineering, and nanotechnology literature not fully indexed in PubMed; Web of Science Core Collection provides coverage of high-impact journals across sports science, biomedical engineering, and physical performance. The complete and exact search strings as executed in all three databases, including field tags, Boolean operators, and search dates, are provided in Supplementary Material Section S1.

The following search string was applied in PubMed/MEDLINE using MeSH terms and free-text fields: (“nanotechnology”[MeSH Terms] OR “nanoparticles”[MeSH Terms] OR “carbon nanotubes”[MeSH Terms] OR “quantum dots”[MeSH Terms] OR “nanosensors”[Title/Abstract] OR “nanomaterials”[Title/Abstract] OR “photobiomodulation”[Title/Abstract] OR “Taopatch”[Title/Abstract]) AND (“athletic performance”[MeSH Terms] OR “exercise”[MeSH Terms] OR “athletes”[MeSH Terms] OR “sports performance”[Title/Abstract] OR “elite athletes”[Title/Abstract] OR “physical performance”[Title/Abstract] OR “exercise performance”[Title/Abstract]).

Equivalent keyword combinations using Title/Abstract/Keyword fields were applied in Scopus and Web of Science, adapted to each database’s controlled vocabulary and field-tag syntax (see Supplementary Material Section S1 for exact strings). The authors acknowledge that additional databases, including SPORTDiscus (sports science literature not indexed in PubMed), Embase (pharmacological and supplementation literature), IEEE Xplore (wearable sensor and materials engineering literature), and discipline-specific engineering and materials science databases, were not searched. This constitutes a notable retrieval limitation with potential impact on coverage of the wearable sensor, materials engineering, and supplementation domains, and is discussed further in Section 4.5. Future iterations of this review must incorporate these databases.

No date limits or language restrictions (beyond the English-language post-screening exclusion criterion) were applied at the database level. No automation tools were used in the screening process. Additional manual searching of reference lists of all included studies and relevant narrative reviews was performed. Grey literature sources, clinical trial registries (ClinicalTrials.gov, EU Clinical Trials Register), and non-English publications were not systematically searched. The absence of grey literature and trial registry searching represents a notable limitation with implications for publication bias, as discussed in Section 4.5; future iterations of this review must include these sources.

### 2.4. Study Selection

**Figure 1:**
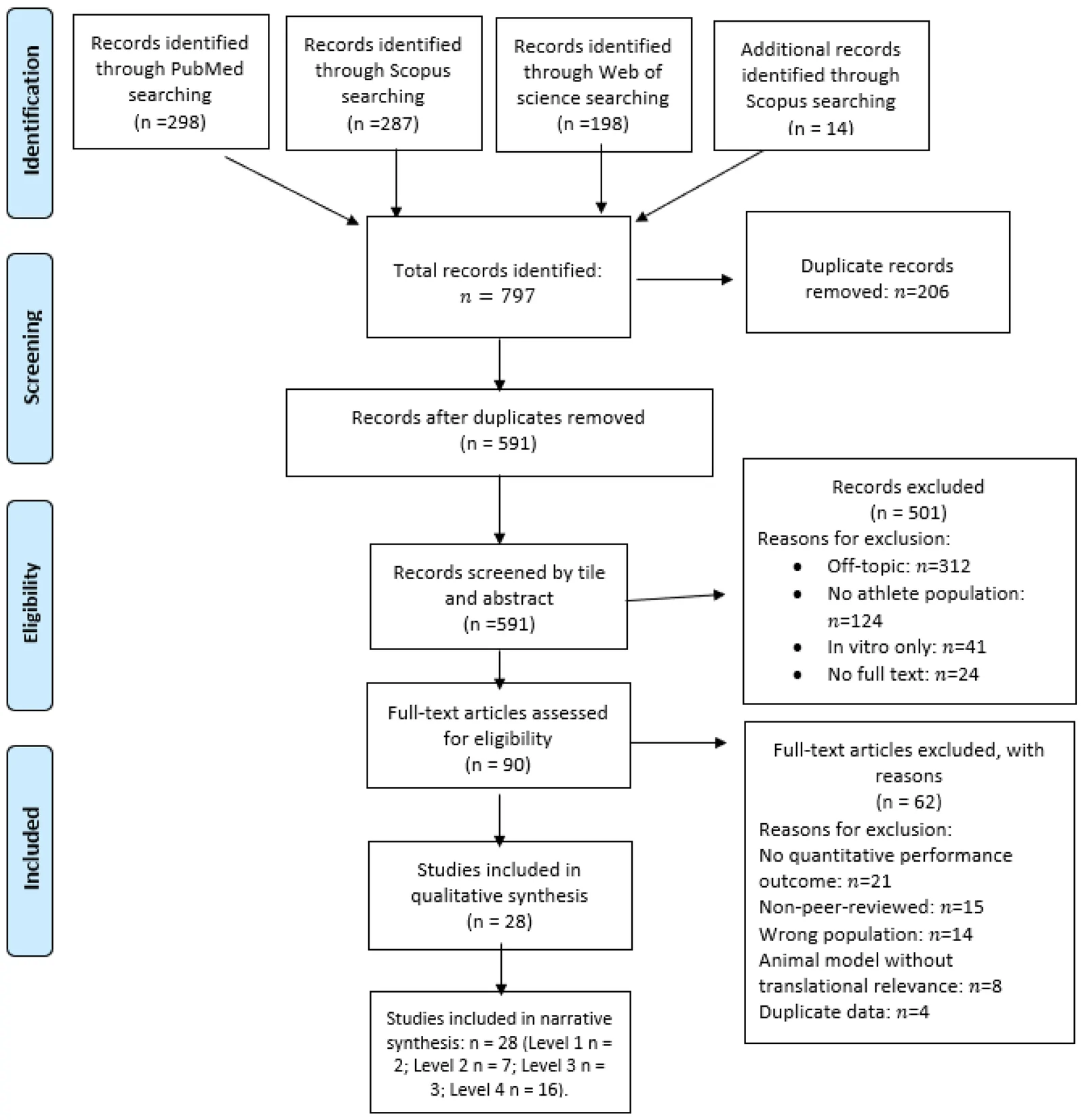
PRISMA-ScR 2020 flow diagram.

Two reviewers (R.D.V. and L.L.) independently screened all titles and abstracts retrieved from the database searches, followed by independent full-text assessment of all potentially eligible records. Disagreements at both stages were resolved by discussion with a third reviewer (M.G.) and, where necessary, by consensus among all authors. The selection process is summarized in the PRISMA-ScR flow diagram (Figure 1).

Inter-rater agreement for both title/abstract screening and full-text eligibility assessment was not formally quantified. Cohen’s κ was not calculated prospectively, and the number of disagreements at each screening stage was not formally recorded as a pre-specified metric. Disagreements were resolved through discussion, with recourse to a third reviewer (M.G.) when consensus was not reached at the two-reviewer level. This constitutes an acknowledged limitation of reproducibility reporting in this review. It should be noted that PRISMA 2020 Item 8 [15] requires reporting of the selection process, including the number of independent reviewers, but does not explicitly mandate calculation of a κ statistic; however, formal quantification of inter-rater agreement is acknowledged as best practice for reproducibility assessment. Future iterations must prospectively record and report percentage agreement and/or Cohen’s κ with 95% CI at both screening stages.

### 2.5. Data Extraction

A standardized data extraction form was prospectively designed and piloted before use. Two reviewers (R.D.V. and L.L.) independently extracted the following variables from each included study: first author; year of publication; country; study design; evidence stratum (1–4); nanotechnological domain; population and sample size; nanotechnological intervention; comparator; outcome measures; key quantitative results; risk of bias or quality assessment score; and authors’ conclusions and stated limitations. Discrepancies in extracted data were resolved by consensus. The data extraction form is available from the corresponding author upon reasonable request and is summarized in Table 3. Sample sizes verified against source publications are reported; where sample size was not retrievable from the accessible source, this is indicated as “NR” in Table 3.

### 2.6. Quality Assessment

Quality appraisal was applied stratum-specifically, in accordance with the evidence strata framework defined in Section 2.2. The quality appraisal tools employed across the four evidence strata are not directly comparable to one another, as each tool is calibrated to a specific study design type; quality appraisal results are therefore presented descriptively within each stratum, and no cross-stratum comparison of quality scores is made. For narrative reviews and materials science studies, the structured narrative quality appraisal frameworks applied are acknowledged methodological compromises that are less structured and less validated than RoB 2, NOS, SYRCLE, or AMSTAR-2; this represents a limitation of the quality appraisal for these study subgroups. The criteria applied in the narrative frameworks are detailed in Supplementary Material Section S5. Risk of bias (a study-stratum methodological quality assessment), certainty of evidence (a GRADE-based synthesis-stratum judgment), and methodological quality (a broader term encompassing study design appropriateness) are not synonymous; all three concepts are used in their specific senses throughout this review. No formal GRADE certainty-of-evidence assessment was performed.

- Level 1: (RCTs and controlled trials): Cochrane Risk of Bias 2 (RoB 2) tool [18], assessing five domains: (1) randomization process; (2) deviations from intended interventions; (3) missing outcome data; (4) measurement of the outcome; and (5) selection of the reported result. Each study was classified with the categorical RoB 2 output: “Low risk of bias,” “Some concerns,” or “High risk of bias” at both domain and overall levels.
- Level 2: (Observational and cross-sectional studies): Newcastle–Ottawa Scale (NOS) [19], with scores from 0 to 9 stars across Selection, Comparability, and Outcome/Exposure domains. Studies scoring ≤4 were classified as high risk of bias; 5–6 as moderate; ≥7 as low.
- Level 3: (Animal studies): SYRCLE’s risk of bias tool for animal intervention studies [20], assessing ten domains adapted from the Cochrane RoB tool.
- Level 4a: True systematic reviews and meta-analyses: AMSTAR-2 [21], providing an overall confidence rating (High, Moderate, Low, or Critically Low) based on 16 items, seven of which are designated as critical domains. AMSTAR-2 was applied exclusively to studies employing formal systematic review methodology (pre-specified search strategy, eligibility criteria, and data synthesis); it was not applied to narrative or comprehensive reviews.
- Level 4a: Narrative and comprehensive reviews: A structured narrative quality appraisal framework was applied, evaluating: (a) clarity of the review question; (b) description of search methods; (c) criteria for inclusion and exclusion of cited evidence; (d) transparency of synthesis methods; and (e) disclosure of conflicts of interest. This approach is acknowledged as less structured than validated tools and represents a limitation of the quality appraisal for this study subgroup.
- Level 4b: (Materials science studies): A narrative quality appraisal was applied, based on the completeness of nanomaterial characterization (spectroscopic, mechanical, and morphological), reproducibility of testing conditions, and relevance of the outcome measures to athletic performance. Standardized tools such as RoB 2 or NOS were not applicable to materials science study designs and were not used; this is explicitly acknowledged as a limitation.

All quality assessments were performed independently by two reviewers (R.D.V. and L.L.) with disagreements resolved by consensus. Results are reported in the full data extraction table (Supplementary Material Section S4) and in the abbreviated evidence map (Table 3, main text), and are summarized narratively in domain-specific sections (Section 3.3). Quality appraisal results influence the synthesis by qualifying the confidence attributable to domain-specific conclusions within each stratum; a domain-stratum synthesis summary (Table 3) integrating study design, directness, aggregate risk of bias classification, consistency, and precision is provided.

### 2.7. Synthesis

Due to substantial heterogeneity across intervention types, populations, study designs, and outcome measures, a narrative synthesis was performed. Meta-analysis was not feasible. The primary sources of heterogeneity included: mechanistic diversity of nanotechnological interventions across six distinct domains; heterogeneity in study design across four evidence strata; variation in performance outcome measures; inconsistent operational definitions of the athlete population; and the inclusion of both primary studies and contextual reviews. These factors collectively precluded the use of a common effect measure and the conduct of meta-analytic synthesis.

Results are presented domain-specifically, with each study’s evidence stratum explicitly labelled throughout. The 28 included records represent evidence sources (publications), not 28 independent bodies of primary evidence; Stratum 4 secondary sources may indirectly cite some of the same primary studies as one another, and this potential for indirect evidence overlap is acknowledged as an inherent limitation. Where included studies are themselves systematic reviews or narrative reviews (Stratum 4), their findings are presented as contextual framework evidence only, clearly distinguished from primary direct or translational evidence (Strata 1–3) throughout. No formal GRADE certainty-of-evidence assessment was performed; domain-stratum characterizations are descriptive and must not be interpreted as formal certainty-of-evidence ratings. In the Discussion, references cited for contextual framing but not included among the 28 primary evidence map records are consistently labeled [contextual reference]; a full list of such references is provided in Supplementary Material Section S2.

## 3. Results

### 3.1. PRISMA-ScR Flow Diagram

The study selection process is summarized in Figure 1. A total of 797 records were identified through database searching and supplementary manual reference list searching. Specifically, 298 records were retrieved from PubMed/MEDLINE (search date: 12 June 2026), 287 from Scopus (search date: 15 June 2026), 198 from Web of Science (search date: 15 June 2026), and 14 through manual searching of reference lists of included studies and relevant narrative reviews. After removal of 206 duplicate records, 591 unique records remained for title and abstract screening.

During the screening phase, 501 records were excluded. The principal reasons for exclusion were off-topic subject matter (n = 312); absence of any athlete or exercise population across all four eligibility strata (n = 124); in vitro-only investigations without in vivo or biomechanical outcome (n = 41); and full text not available (n = 24). Subsequently, 90 full-text articles were assessed for eligibility across all four strata.

Of these, 62 full-text articles were excluded for the following reasons: absence of quantitative performance or exercise-relevant physiological outcome (n = 21); non-peer-reviewed publication type (n = 15); study population outside all four eligibility strata (n = 14); animal model in a domain other than nano-supplementation (n = 8); and duplicate data from a previously included study (n = 4).

Ultimately, 28 studies met eligibility criteria across the four evidence strata and were included in the narrative synthesis. The evidence stratum distribution was as follows: Stratum 1 (direct RCTs/controlled trials in athletes or healthy subjects), n = 2; Stratum 2 (observational/quasi-experimental in humans), n = 7; Stratum 3 (animal exercise models), n = 3; Stratum 4 (systematic reviews, meta-analyses, narrative reviews, and materials science studies; subdivided into Stratum 4a, n = 12, and Stratum 4b, n = 4), n = 16.

### 3.2. Characteristics of Included Studies

The 28 included records are organized into two explicitly distinct evidence streams: (1) primary experimental evidence, comprising Strata 1–3 (n = 12 studies: 2 controlled human trials, 7 observational/quasi-experimental human studies, 3 animal exercise studies); and (2) secondary and contextual framework evidence, comprising Stratum 4 (n = 16 records: 5 true systematic reviews/meta-analyses, 7 narrative/comprehensive reviews, 4 materials science studies). These two streams serve different roles in the evidence map: Strata 1–3 records provide direct or translational data on performance-relevant outcomes; Stratum 4 records provide technical, mechanistic, and contextual framing. Stratum 4 records should not be interpreted as independent primary evidence and may share primary study citations with one another. The abbreviated evidence map is presented in Table 3 below; the full data extraction table is available as Supplementary Material Section S4.

### 3.3. Domain-Specific Findings

The following domain-specific sections apply a three-stage interpretive framework to each domain: (1) technological feasibility (evidence from materials science or technical validation studies); (2) physiological or biomechanical validation in human subjects; and (3) demonstrated athletic performance benefit in trained or elite athletes. This framework is applied consistently to distinguish what the current evidence shows from what it does not yet demonstrate. Each domain section explicitly labels the evidence stratum of each cited study.

#### 3.3.1. Carbon Nanotube-Based Sports Equipment

(Stratum 4b: Materials science and contextual reviews; Stratum 1: one controlled human study)

Carbon nanotubes are allotropes of carbon with a cylindrical nanostructure, exhibiting an elastic modulus of 1–5 TPa and tensile strength approximately 100 times that of steel at one-sixth the weight [4]. CNTs have been integrated into tennis racquets, bicycle frames, baseball bats, snowboards, running shoes, and carbon-fiber composites across multiple sports disciplines [2,5].

A Stratum 4b peer-reviewed systematic analysis of CNT-reinforced polymer composites demonstrated consistent improvements in Young’s modulus, tensile strength, and wear resistance with increasing CNT content, including a 50% improvement in wear resistance in block-on-ring testing [4]. Harifi and Montazer reviewed applications of nanotechnology in sports clothing, flooring, and equipment, reporting that CNT-reinforced structures may improve deformation resistance and functional textile properties in sports contexts [3]. A subsequent review of CNT-reinforced polymer composites demonstrated consistent improvements in Young’s modulus, tensile strength, and wear resistance with increasing CNT content [4]. Miao further examined the relationship between conjugated nanomaterial processing and sports equipment design, reporting that CNT- and graphene-based composites may optimize stiffness-to-weight ratio and energy-transfer efficiency in sports equipment models [5].

The only Stratum 1 controlled human study in this domain, Ko et al. [17], demonstrated that carbon-fiber insoles improved vertical jump performance by 1.5% and agility performance by 1.0% compared to conventional insoles in healthy males (*p* < 0.05). This study is classified as Stratum 1 based on its controlled crossover design; however, the enrolled population comprised healthy adult males not formally classified as elite athletes, positioning this study at the lower boundary of Stratum 1. Its evidence should be interpreted as proof-of-concept controlled human evidence, not direct elite athlete evidence. The study employed a within-subject acute crossover design; sample size calculations and MCID thresholds were not reported [17]. The 1.5% improvement in vertical jump height was statistically significant but should be interpreted with caution in the absence of a reported MCID threshold or smallest worthwhile change (SWC) calculation; statistical significance does not in itself confirm practical performance relevance in an elite athletic context. Translational gap summary for this domain: (1) technological feasibility, established in materials science literature [3,4,5]; (2) physiological/biomechanical validation in humans, limited proof-of-concept evidence from one Stratum 1 crossover study in healthy non-elite males [17]; (3) demonstrated athletic performance benefit in trained/elite athletes, absent from the current literature. The carbon-fiber insole represents a macro-composite rather than a purely nanoscale intervention; its inclusion reflects the continuum between nano- and macro-composite material engineering, and this classification is explicitly stated. The RoB 2 assessment yielded an overall classification of “Some concerns,” with concerns identified in the domains of blinding of outcome assessors (D4) and selective outcome reporting (D5).

The Stratum 4b evidence is predominantly derived from laboratory mechanical testing and computational modelling. The extrapolation from improved material properties (e.g., Young’s modulus, tensile strength) to quantifiable competitive performance gains in elite athletes represents an inferential step that, while mechanistically plausible, has not yet been formally validated in adequately powered prospective athletic trials. The absence of in-competition elite athlete performance data in the peer-reviewed literature remains the principal translational gap in this domain.

#### 3.3.2. Nanoparticle-Based Antioxidant Supplementation and Nutrient Delivery

(Stratum 3: Animal exercise models; Stratum 4a: Contextual reviews)

Exercise-induced oxidative stress, characterized by the overproduction of reactive oxygen species (ROS), is a well-established physiological challenge in elite sport, contributing to muscle damage, impaired recovery, and performance decrements [36]. Conventional antioxidant supplementation is limited by poor bioavailability and by the paradoxical impairment of training adaptation at high doses [36]. Nanoparticle encapsulation strategies may address some of these limitations through improved bioavailability, targeted delivery, and controlled-release kinetics, although direct confirmation in elite athletes remains unavailable.

All three primary studies in this domain are classified as Stratum 3 (animal exercise models). Toriumi et al. (2021) [6] administered a pH-sensitive self-assembling antioxidant nanoparticle (RNPN) subcutaneously to Wistar rats (n ≈ 7 per group), extending treadmill running time by approximately 50% (from 60 to 90 min at 40 m/min) compared to controls, with significantly attenuated post-exercise reduction in circulating red blood cells (RBCs), indicating protection against exercise-induced oxidative hemolysis. This study represents dual translational barriers: a rodent-to-human species extrapolation, and a subcutaneous administration route with no clinically realistic equivalent for athletes. The priority claim made by the authors of reference [6], characterizing this as the first nanoparticle-type antioxidant study in exercise performance, should be treated as the authors’ own assertion and has not been independently corroborated.

A subsequent study by Toriumi et al. (2023) [7] using orally administered ROS-scavenging nanoparticles (RNPO) selectively targeting the gastrointestinal (GI) tract in C57BL/6J mice (n = 7–8 per group) demonstrated significantly extended running time to exhaustion in a dose-dependent manner, in contrast to the conventional antioxidant TEMPOL, which reduced running time at higher doses. Nanoparticle-treated mice exhibited protected GI mucosa and significantly lower plasma lipopolysaccharide (LPS) levels post-exercise, providing a mechanistic basis for nano-antioxidant efficacy through protection against exercise-induced gastrointestinal syndrome (EIGS). Resveratrol-loaded solid lipid nanoparticles (RSV-SLNs) were evaluated in vivo in ICR mice (n = 10 per group) by Qin et al. [8], demonstrating prolonged exhaustive running time and improved antioxidant biomarkers (superoxide dismutase [SOD], malondialdehyde [MDA]) compared to free resveratrol, with the SIRT1/autophagy pathway (LC3-II, p62) identified as a mechanistic mediator.

All three Stratum 3 studies [6,7,8] were conducted exclusively in rodent models. Neither study enrolled human participants. The translational applicability to elite athletes is therefore speculative at the current evidence level, and these findings must be interpreted accordingly. SYRCLE risk of bias assessment yielded “unclear” ratings across multiple domains for all three studies, primarily due to insufficient reporting of sequence generation, allocation concealment, and blinding of outcome assessors, which is consistent with the broader animal exercise literature.

#### 3.3.3. Wearable Nanosensors and Real-Time Physiological Monitoring

(Stratum 2: Validated human sensor studies; Stratum 4a: Contextual reviews and technical studies)

The integration of nanomaterials, particularly CNTs, graphene, and conductive nano-composites, into flexible wearable sensors represents a significant advance in athlete monitoring technology [9,10].

Wyckoff et al. (Stratum 2) [24] developed and validated multi-walled CNT (MWCNT) elastic fabric motion tape sensors for low back movement characterization in 20 participants (n = 10 per material group), demonstrating that MWCNT-based sensors exceeded graphene nanosheet equivalents in signal stability under high-strain conditions and accurately characterized forward flexion, lateral bending, and rotation, confirming their applicability to clinical and sports biomechanical monitoring contexts. NOS assessment yielded a high-quality score of 8★/9. Seshadri et al. (Stratum 4a, narrative review) [9] reviewed wearable sensor technologies for monitoring internal and external athletic workloads, identifying nanomaterial-based electrochemical sensors, capable of real-time sweat lactate, glucose, and electrolyte monitoring, as the most promising emerging technology for non-invasive performance physiology. It should be noted that Seshadri et al. [9] is a narrative review; AMSTAR-2 was not applied, and a structured narrative quality appraisal was used instead. Pirzada and Altintas (Stratum 4a, narrative review) [10] documented that CNT- and graphene-based transducers achieve detection limits several orders of magnitude lower than conventional electrode materials, with high selectivity for metabolites directly relevant to athletic monitoring.

#### 3.3.4. Photobiomodulation Nanotechnology: Taopatch^®^ Devices

(Stratum 1: One small RCT; Stratum 2: Observational and quasi-experimental studies; Stratum 4a: Contextual PBM systematic reviews)

Three critical preliminary statements are required before this domain’s evidence is presented:

- First, conventional photobiomodulation therapy (PBMT) using low-level laser therapy (LLLT) or LED devices is not itself a nanotechnological intervention. Evidence from conventional PBMT systematic reviews [29,30,31,32] is presented in Section Evidence for Conventional PBM Efficacy (Stratum 4a—Contextual Framework Evidence Only) as contextual framework evidence establishing the biological plausibility of photobiomodulation as a device category; it does not constitute evidence of efficacy for Taopatch^®^ or any nanotechnology-enabled PBM device
- Second, evidence specifically pertaining to Taopatch^®^ is presented separately in Section Evidence Specific to Nanotechnology-Enabled PBM Devices (Taopatch^®^)—Strata 1–2 and must be evaluated on its own merits, which are limited by the methodological concerns detailed below.
- Third, the following three caveats are reiterated here for interpretive clarity: (a) the nanomaterial composition of Taopatch^®^ has not been independently characterized in any peer-reviewed spectroscopic or nanotechnology publication; (b) the photon emission characteristics and delivered irradiance have not been independently quantified; (c) CE Class I marking reflects conformity with general safety and performance requirements, not regulatory demonstration of clinical efficacy.

##### Evidence for Conventional PBM Efficacy (Stratum 4a—Contextual Framework Evidence Only)

Four Stratum 4a systematic reviews and meta-analyses provide contextual support for photobiomodulation as a device category [29,30,31,32]. Dutra et al. [31] demonstrated significant ergogenic effects of PBMT on mode-specific exercise performance across 37 studies (muscle endurance SMD 0.27, 95% CI 0.12–0.41; cycling time to exhaustion SMD 0.35, 95% CI 0.10–0.59); De Marchi et al. [32] confirmed that PBMT significantly attenuates exercise-induced oxidative stress; Leal-Junior et al. [30] identified pre-exercise irradiation at 660–905 nm, 20–50 J per muscle group, as the parameter range yielding the most consistent performance benefits; and Vanin et al. [29] confirmed significant improvements in muscular performance and reduction of fatigue across 39 RCTs. These findings establish that photobiomodulation, when delivered by conventional PBMT devices at characterized therapeutic wavelengths and irradiances, has a consistent ergogenic effect supported by moderate-confidence systematic review evidence. However, this body of evidence cannot be applied to Taopatch^®^ unless the device’s emission characteristics are independently confirmed to match those of validated PBMT devices, which has not occurred.

##### Evidence Specific to Nanotechnology-Enabled PBM Devices (Taopatch^®^)—Strata 1–2

Taopatch^®^ (Tao Technologies Srls, Cittadella, Italy) is a CE Class I medical device claimed by the manufacturer to emit light across 400–1400 nm when activated by body-heat-generated infrared radiation, with proposed modulation of cutaneous photoreceptors and proprioceptive afferents through quantum confinement-analogous optical principles. The following findings must be interpreted in light of the three caveats stated above.

Malchiodi Albedi et al. [11] conducted a small parallel-group RCT (n = 40, healthy subjects; Stratum 1) evaluating Taopatch^®^ devices on postural control using stabilometry. The nanotechnology device group showed statistically significant improvement in postural control parameters versus sham (*p* < 0.05). RoB 2 assessment yielded an overall classification of “High risk of bias,” with concerns identified in: D1 (randomization process not fully described); D2 (feasibility of sham blinding not documented); D4 (outcome assessor blinding insufficient); and D5 (no pre-registered protocol; selective reporting cannot be excluded). The population comprised healthy subjects, not elite athletes. The authors appropriately qualified these as preliminary data.

Carbonari et al. [22] conducted a retrospective cross-over study (n = 25 athletes; Stratum 2) evaluating Taopatch^®^ versus occlusal splint effects on muscular performance, balance, and posture. NOS assessment yielded a score of 4★/9 (high risk of bias), reflecting the retrospective, unblinded, non-randomized design. The observation that both Taopatch^®^ and an unrelated mechanical intervention (occlusal splint) produced measurable improvements raises a strong possibility of a placebo effect, which cannot be excluded without a true sham control group.

Messina et al. [12] conducted a prospective observational study in multiple sclerosis (MS) patients (n = 20; not elite athletes; Stratum 2) demonstrating statistically significant improvements in baropodometric measures (*p* < 0.05) and handgrip strength bilaterally (right *p* = 0.023; left *p* = 0.021) following Taopatch^®^ application combined with a proprioceptive rehabilitation protocol, with a trend to increased vitamin D levels that did not reach statistical significance. NOS score: 4★/9. Generalization to elite athletes is not appropriate without further specific evidence. The mechanistic pathway by which a topically applied PBM device would produce a measurable increase in circulating 25-hydroxyvitamin D remains unexplained and requires independent replication before any causal interpretation is made.

Campoli et al. [25] conducted a pilot quasi-experimental study evaluating a Taopatch^®^-integrated hearing aid device in 40 hearing aid users (Stratum 2, non-athletic population, non-randomized in the nanotechnology allocation dimension) demonstrating preliminary positive effects on posturographic balance parameters (NOS 3★/9). The combination of Taopatch^®^ with a hearing aid introduces a multimodal intervention structure that prevents attribution of any observed effect to the nanotechnological component specifically; this constitutes an unresolved confounding issue.

Amato et al. [23] conducted a pilot study in 10 MS patients during the COVID-19 pandemic (Stratum 2, non-athlete population, NOS 3★/9) retained for completeness given its mechanistic relevance to Taopatch^®^ evidence, and explicitly labelled throughout as a non-athlete, non-sport population study.

Translational gap summary for this domain: (1) technological feasibility, not independently established in peer-reviewed nanotechnology literature; (2) physiological/biomechanical validation in humans, limited, with signals of possible effect on postural control parameters in small studies with high risk of bias; (3) demonstrated athletic performance benefit in trained/elite athletes, absent.

Overall assessment (Taopatch^®^-specific evidence only): Available evidence from Strata 1–2 is insufficient to support any conclusion, positive or negative, regarding the efficacy of Taopatch^®^ for elite athletic performance improvement. The current Taopatch^®^-specific evidence is characterized by small sample sizes, high risk of bias in all controlled studies, predominantly non-athletic and clinical populations, absence of pre-registered protocols, and unresolved mechanistic and characterization questions. The convergence of contextual evidence from conventional PBMT systematic reviews (Section Evidence for Conventional PBM Efficacy (Stratum 4a—Contextual Framework Evidence Only)) supports PBM as a device category but cannot be directly extrapolated to Taopatch^®^ unless the device’s emission spectrum and irradiance are independently confirmed. Well-designed, adequately powered, double-blind, placebo-controlled RCTs in elite athletes, incorporating independent nanomaterial characterization and spectroscopic dosimetry verification, pre-specified outcomes, and a verified sham control condition, are necessary before any clinical recommendation can be formulated.

It is noteworthy that the included studies report divergent emission ranges for Taopatch^®^ (200–600 nm, 200–900 nm, and 400–1400 nm), further corroborating the absence of standardized and independent spectroscopic characterization of the device. 

Four Stratum 4a systematic reviews and meta-analyses provide contextual support for photobiomodulation as a device category [29,30,31,32]. Dutra et al. [31] demonstrated significant ergogenic effects of PBMT on mode-specific exercise performance across 37 studies (muscle endurance SMD 0.27, 95% CI 0.12–0.41; cycling time to exhaustion SMD 0.35, 95% CI 0.10–0.59); De Marchi et al. [32] confirmed that PBMT significantly attenuates exercise-induced oxidative stress; Leal-Junior et al. [30] identified pre-exercise irradiation at 660–905 nm, 20–50 J per muscle group, as the parameter range yielding the most consistent performance benefits; and Vanin et al. [29] confirmed significant improvements in muscular performance and reduction of fatigue across 39 RCTs.

#### 3.3.5. Quantum Dots: Biomedical Applications and Sports-Relevant Biosensing

(Stratum 4a: Contextual biomedical and engineering reviews; no Stratum 1–3 athlete study exists)

Quantum dots are semiconductor nanocrystals (1–10 nm diameter) exhibiting quantum confinement effects, conferring size-tunable fluorescence emission, high quantum yield, broad excitation spectra, and exceptional photostability compared to conventional organic fluorophores [13,14]. Narrative reviews [13,14,34] document QD applications in in vitro diagnostics, molecular imaging, drug delivery, and theranostics, with over 10,000 peer-reviewed publications on QD biomedical applications. These reviews were appraised using a structured narrative quality framework; AMSTAR-2 was not applied. Principal challenges to clinical translation include the cytotoxicity of heavy-metal-containing QDs (CdSe, CdTe) and limited in vivo clearance data [27].

Mansuriya and Altintas [37] demonstrated that graphene quantum dot (GQD)-based electrochemical immunosensors achieve femtomolar detection limits for cytokines and metabolic biomarkers of direct relevance to athlete physiology. Li et al. (2023) [27] demonstrated that heavy-metal-free Cu-In-Se QD-based photodetectors enable high-fidelity photoplethysmography (PPG) for wearable cardiovascular monitoring, a direct sports-relevant application for heart rate and blood oxygen saturation monitoring, in a human validation study (n = 5). No published peer-reviewed study has applied conventional QDs or GQDs directly to elite athlete populations for performance monitoring or improvement in vivo. All current evidence derives from materials science, biomedical engineering, or non-sport clinical contexts. QD applications to sport performance monitoring remain at an early and speculative stage of research.

### 3.4. Cross-Domain Translational Considerations

Beyond domain-specific findings, the systematic literature search identified a series of cross-cutting studies with direct translational relevance, presented here to ensure completeness and sequential citation order.

Regarding CNT biocompatibility, Abdellatif et al. [38] comprehensively reviewed QD cytotoxicity, confirming that cadmium-based QD formulations (CdSe, CdTe) present significant dose-dependent cytotoxicity concerns and limited in vivo clearance data, the primary barrier to clinical QD translation, while confirming that heavy-metal-free alternatives are in active development. Nomura et al. [39] documented dose-dependent granulation tissue formation without sustained chronic inflammation in the synovial membrane response to multi-walled carbon nanotubes (MWCNTs), with direct relevance to the long-term safety evaluation of CNT-composite sports equipment. Li et al. [40] reviewed conjugated nanomaterial-based sports rehabilitation materials, supporting the potential of optimized nano-composite configurations to enhance mechanical and functional properties of both protective and rehabilitative sports equipment. Akiyama et al. [26] demonstrated that an externally applied, natural-mineral-based novel nanomaterial (IFMC) produced measurable improvements in cardiopulmonary function parameters, including oxygen uptake and cardiac output indices, during aerobic exercise (Stratum 2: human subjects, acute exercise study), providing preliminary human evidence of performance-relevant physiological effects attributable to a non-invasive nanomaterial application.

As an evidence benchmark for nano-encapsulated variants, Fernandes et al. [41] systematically reviewed conventional CoQ10 supplementation in athletes across multiple RCTs, demonstrating capacity to reduce oxidative stress markers and creatine kinase levels while improving anaerobic performance at oral doses of 30–300 mg; nanoparticle encapsulation of CoQ10 represents a rational strategy to further enhance these effects by circumventing the bioavailability limitations inherent to its lipophilic molecular structure. Mansuriya and Altintas [42] established the analytical foundation for GQD-based athlete monitoring by demonstrating femtomolar detection limits for cytokines and metabolic biomarkers via GQD electrochemical immunosensors. Yamada et al. [35] validated stretchable single-walled carbon nanotube (SWCNT)-based strain sensors for high-fidelity human motion detection across a wide range of strain (up to 280%), with direct applicability to athlete biomechanical monitoring. Fathy and Bühlmann [33] reviewed next-generation flexible and wearable potentiometric sensor architectures, including CNT-based solid-contact ion-selective electrodes (ISEs) and nanoporous carbon transducers for multi-analyte sweat monitoring (Na^+^, K^+^, pH, NH_4_^+^), confirming athlete sweat analysis as a primary application domain with multiple wearable potentiometric systems validated in exercise contexts.

## 4. Discussion

The following discussion integrates the mapped evidence with selected contextual literature on sports biomechanics, antioxidant bioavailability, wearable sweat monitoring, rehabilitation nanomaterials, and nanoparticle pharmacology. These contextual references are used to interpret translational relevance and were not counted among the 28 studies included in the primary evidence map unless explicitly listed in Table 3.

### 4.1. Summary of Principal Findings

The following domain-stratum characterizations reflect the relative maturity of the technical evidence base for each nanotechnological domain. They are descriptive in nature and do not constitute formal certainty-of-evidence ratings; no GRADE assessment was performed. A domain-stratum evidence summary matrix is provided as Table 4, separating technical feasibility, human physiological/biomechanical validation, direct athletic performance evidence, and aggregate risk of bias classification for each domain.

#### 4.1.1. Most Technically Advanced and Reproducible Evidence Base

At the technological feasibility stage, CNT-reinforced equipment composites demonstrate consistent and reproducible improvements in strength-to-weight ratio, wear resistance, and energy transfer characteristics in the materials science literature [4,5] (Stratum 4b). At the physiological/biomechanical validation stage, a single Stratum 1 crossover study [17] provides limited proof-of-concept evidence in healthy non-elite males. At the demonstrated athletic performance benefit stage, no adequately powered prospective trial in trained or elite athletes has been conducted.

The only controlled human study, Ko et al.’s carbon-fiber insole crossover trial [17], provides proof-of-concept data (1.5% jump height improvement, *p* < 0.05) from a population of healthy non-elite males with a “Some concerns” RoB 2 overall rating. This improvement was statistically significant; however, in the absence of a reported MCID threshold or SWC calculation for this outcome in this population, the practical performance significance remains uncertain and should not be overstated. The extrapolation from improved material properties to quantifiable competitive performance gains in elite athletes remains a mechanistic hypothesis, not a demonstrated outcome. This observation aligns with the broader principle of marginal gains in elite sport, whereby incremental biomechanical optimizations accumulate into meaningful competitive advantages [3]. The same biomechanical rationale has been extended to peripheral upper-limb interventions in soccer: De Vitis et al. [43] recently demonstrated, in a scoping review, that hand-wrist bandaging may improve kicking performance by optimizing arm-swing mechanics and neuromuscular coordination, underscoring how peripheral biomechanical factors previously considered marginal can exert quantifiable effects on elite athletic output.

The extrapolation from improved material properties (Young’s modulus, tensile strength) to quantifiable competitive performance gains in elite athletes remains an inferential step that, while mechanistically plausible, has not yet been validated in adequately powered prospective athletic trials.

#### 4.1.2. Consistent Preclinical Evidence, Absent Human Athlete Data

At the technological feasibility and translational animal evidence stage, nanoparticle-based antioxidant strategies demonstrate significant improvements in running time to exhaustion and protection against exercise-induced systemic damage in Stratum 3 rodent models [6,7]. At the physiological/biomechanical validation stage, no controlled human data exist. At the demonstrated athletic performance benefit stage, no data exist. These findings must be interpreted as translational animal evidence only; their applicability to elite athletes remains speculative. RSV-SLNs similarly outperform free-form resveratrol in anti-fatigue outcomes [8]. The critical translational gap from animal model to human elite athlete remains to be bridged. A fundamental limitation of resveratrol as a conventional supplement in humans is its extremely poor oral bioavailability due to rapid first-pass hepatic metabolism; nanoparticle encapsulation directly addresses this pharmacokinetic barrier [44], making nano-formulated delivery a qualitatively, not merely incrementally, superior translational approach. CoQ10, a further nano-encapsulable antioxidant of direct relevance to mitochondrial bioenergetics, has demonstrated in a human athlete systematic review [41] the capacity to reduce oxidative stress markers and creatine kinase levels while improving anaerobic performance at oral doses of 30–300 mg; nanoparticle encapsulation of CoQ10 represents a rational strategy to amplify these effects by circumventing the bioavailability limitations of its lipophilic molecular structure [41].

A recent GRADE-assessed dose–response meta-analysis of CoQ10 supplementation further supports the relevance of dose and formulation when interpreting antioxidant effects on exercise-induced muscle damage and performance [45].

A 2025 PRISMA-compliant systematic review and meta-analysis of 26 RCTs involving 505 athletes [46] demonstrated that conventional antioxidant supplementation significantly reduced post-exercise lactate (SMD: −1.25; 95% CI: −2.06, −0.43) and creatine kinase levels (SMD: −1.88; 95% CI: −2.98, −0.78), while failing to produce statistically significant overall effects on MDA, SOD, and GPx, with substantial interstudy heterogeneity and a high risk of bias in the included studies. This pattern of partial, heterogeneous efficacy with conventional formulations reinforces the translational rationale for nano-antioxidant strategies. It should be noted, however, that enhanced bioavailability could theoretically amplify the paradoxical impairment of training adaptation documented with high-dose antioxidants [36]; this represents an additional translational complexity requiring investigation in human studies before nano-antioxidant supplementation can be recommended in elite athletes [36].

#### 4.1.3. Technically Validated; Human Physiological Evidence Exists; Direct Athletic Performance Benefit Not Yet Demonstrated

Wearable nanosensor technologies demonstrate technical feasibility (Stratum 4 materials science and engineering evidence [4,5,33,35]) and human physiological/biomechanical validation in non-elite subjects (Stratum 2 [24]). Direct demonstration of performance benefit in trained or elite athletes from wearable nanosensor application has not been reported in the peer-reviewed literature to date. A 2024 comprehensive review [47] identified glucose, lactate, electrolytes, pH, and cortisol as primary target analytes, confirming nanomaterial-based electrochemical transducers as the most sensitive and flexible platforms for non-invasive athlete physiological assessment. Specifically, wearable devices for continuous sweat lactate monitoring have progressed significantly in analytical range (0–100 mM), real-time data transmission, and deformation tolerance during physical activity, though the quantitative relationship between sweat lactate and blood lactate concentrations remains an unresolved translational challenge [48]. Niazi et al. [49] synthesized preclinical evidence for nanomaterial-mediated enhancement of musculoskeletal repair and neuromuscular activation across multiple physical modalities, while explicitly acknowledging that clinical validation in humans remains limited and that translation is constrained by safety uncertainties and regulatory barriers; this reinforces the biological plausibility of nanotechnology-mediated enhancement of tissue responses to physical stimuli.

#### 4.1.4. Preliminary Evidence with Unresolved Mechanistic and Characterization Questions

Taopatch^®^ devices have produced signals of possible effect on postural control in small, heterogeneous Stratum 1 and Stratum 2 studies [11,22,23,25]. No adequately powered, double-blind RCT in elite athletes exists. The claimed emission of Taopatch^®^ across 400–1400 nm encompasses a range far broader than the established therapeutic optical window (approximately 600–1100 nm) of validated PBMT protocols; the irradiance delivered by body-heat activation to underlying tissues has not been quantified in any peer-reviewed study. Evidence from conventional PBMT device trials must not be interpreted as evidence of efficacy for the specific nanotechnology device. The biological mechanism, while plausible within established PBM frameworks, requires independent nanomaterial characterization and controlled mechanistic investigation. Four independent Stratum 4a systematic reviews provide important contextual support for photobiomodulation as a device category [29,30,31,32]: Dutra et al. [31] demonstrated significant ergogenic effects of photobiomodulation therapy (PBMT) on mode-specific exercise performance, including cycling power output, running time to exhaustion, and muscular strength, across 37 studies; De Marchi et al. [32] confirmed that PBMT significantly attenuates exercise-induced oxidative stress markers while enhancing endogenous antioxidant enzyme activity; Leal-Junior et al. [30] specified that pre-exercise irradiation at optimal parameters (660–905 nm, 20–50 J per muscle group) yields the most consistent performance benefits; and Vanin et al. [29] confirmed that PBMT significantly improves muscular performance and reduces exercise-induced fatigue when applied immediately before exercise across 39 RCTs.

The convergence of these four independent Stratum 4a systematic reviews [29,30,31,32] supports PBM as an ergogenic and anti-fatigue modality at the device-category level, reinforcing the biological plausibility of nanotechnology-based PBM devices when such devices deliver photons at characterized therapeutic wavelengths and irradiances. However, the inference that validated ergogenic effects of conventional PBMT devices extend to Taopatch^®^ requires independent spectroscopic and dosimetric confirmation. The claimed emission of Taopatch^®^ across 400–1400 nm encompasses a range far broader than the therapeutic optical window (typically 600–1100 nm) used in validated PBMT protocols, and the irradiance delivered by body-heat activation to underlying tissues has not been quantified in any peer-reviewed study.

An earlier meta-analysis by Leal-Junior et al. [28], focusing on phototherapy effects (low-level laser therapy [LLLT] and LED therapy) on exercise performance and recovery markers, established that pre-exercise phototherapy produces consistent, replicable improvements in muscle fatigue resistance and post-exercise recovery, providing foundational evidence upon which nanotechnology-based photoemission systems should be benchmarked.

#### 4.1.5. Theoretical Stage Only

Direct application of QDs to elite athletic performance improvement in vivo does not yet exist in the peer-reviewed literature. QD-based wearable health monitoring represents the most proximate sports-relevant application, with Li et al. [27] demonstrating technical feasibility for PPG-based cardiovascular monitoring using heavy-metal-free Cu-In-Se QD photodetectors.

### 4.2. Mechanistic Framework

The following mechanistic axes represent plausible biological frameworks for understanding nanotechnology-mediated performance enhancement. They should not be interpreted as demonstrated pathways of efficacy in elite athletes; each axis involves mechanistic hypotheses that remain to be confirmed in adequately powered human athletic trials. Biomechanical/mechanical axis: CNT composites reduce equipment mass, increase stiffness at a given weight, and optimise energy return, directly reducing the energetic cost of athletic movement and improving force transmission [4,5]. This axis extends to nano-composite materials integrated into sportswear and protective gear, which can modify proprioceptive feedback and neuromuscular coordination patterns, biomechanically analogous to other peripheral performance-modulating interventions including upper-limb bandaging strategies in kicking sports [43].

Biochemical/metabolic axis: Nano-encapsulated antioxidants and bioactives address exercise-induced oxidative stress with superior pharmacokinetic profiles, protecting structural tissues (RBCs, GI mucosa, skeletal muscle) and potentially extending exercise capacity [6,7]. The bioavailability advantage conferred by nanoparticle encapsulation is particularly critical for hydrophobic compounds such as resveratrol and CoQ10, for which conventional oral delivery yields systemic concentrations insufficient to reproduce the anti-fatigue effects documented in vitro and in animal models [41,44]. High-dose conventional antioxidants may paradoxically impair training adaptation [36], a limitation that nanoparticle-based targeted delivery may help circumvent, though this must be confirmed in human studies.

Neurophysiological axis: PBM-active nanotechnological devices (Taopatch^®^) hypothetically modulate cutaneous photoreceptors and proprioceptive pathways, potentially improving neuromuscular coordination, postural stability, and movement economy [11,12,22]. The neurophysiological plausibility of this axis is supported by independent PBMT literature demonstrating pre-exercise phototherapy modulation of mitochondrial function through cytochrome c oxidase photoactivation and nitric oxide photodissociation [30,31]. This mechanistic framework is explicitly applicable to Taopatch^®^ only to the extent that the device delivers photons to target tissues at therapeutic wavelengths and irradiances, which has not been independently confirmed.

### 4.3. Comparison with Existing Literature

The most comprehensive recent narrative overview of nanotechnology in sport [2] corroborates the present findings, identifying wearable sensors, functional fabrics, and sports medicine applications as the most developed domains. The present systematic scoping review adds methodological rigor, explicit quality appraisal stratified by evidence stratum, a stratified eligibility framework that accommodates the full breadth of the evidence base, and a specific focus on translational implications for elite athlete populations.

Wang and colleagues (2026) [50] synthesized preclinical and mechanistic evidence suggesting that exercise-induced physiological changes, including increased skeletal muscle blood flow, transient increases in cell membrane permeability, and upregulation of membrane transporters, may create a physiologically receptive environment hypothetically enhancing nanoparticle tissue distribution and cellular uptake. The authors explicitly framed these mechanisms as potentially complementary rather than established, and their clinical relevance in healthy athlete populations has not been directly investigated. These findings should be interpreted as mechanistic hypothesis generation only. The authors explicitly framed these mechanisms as potentially complementary rather than established, and their clinical relevance in healthy athlete populations has not been directly investigated. Importantly, enhanced nanoparticle cellular uptake during exercise carries implicit safety implications, particularly for CNT-containing or heavy-metal-based QD formulations, and should be formally evaluated in safety studies before exercise-concomitant nanoparticle administration is proposed as a strategy.

The absence of any robust RCT exclusively enrolling elite athletes across any nanotechnological domain is the field’s most significant current limitation. Ethical considerations regarding athlete welfare in experimental protocols, access to elite athlete cohorts, and heterogeneous performance metrics across sports disciplines all contribute to this gap.

### 4.4. Safety and Ethical Considerations

#### 4.4.1. Toxicological Profile

CNTs exhibit cytotoxic and genotoxic potential in vitro and in vivo, particularly when inhaled or deposited in biological tissues [39]. The toxicological profile of CNT-reinforced composites in sports equipment is substantially different from that of free CNT fibers: in intact composite form, CNTs are embedded within a polymer matrix and are not expected to be bioavailable under normal use conditions. The primary toxicological concern arises during manufacturing, machining, or end-of-life disposal, where aerosolization of CNT fibers may occur. Manufacturers and sporting goods regulators should develop and publish CNT-composite safety guidance covering the full product lifecycle in accordance with EU REACH provisions applicable to advanced materials.

Heavy-metal-containing QDs (CdSe, CdTe) have recognised cadmium toxicity concerns; heavy-metal-free alternatives (Cu-In-Se, graphene QDs) are in active development and represent the appropriate direction for sports-relevant biosensing applications [27,38].

#### 4.4.2. Anti-Doping Regulations

The World Anti-Doping Agency (WADA) Prohibited List (2024) does not currently include any nanotechnological materials or devices. However, nano-encapsulated delivery systems for otherwise prohibited substances would retain their prohibited status. Athletes and physicians are advised to consult the current WADA Prohibited List (www.wada-ama.org) before employing any nanotechnological intervention. Novel nano-antioxidant formulations may not be detectable by current anti-doping testing methodologies; specifically, nanoparticle-encapsulated permitted compounds (resveratrol-SLN, CoQ10-NP) may alter pharmacokinetic profiles in ways that affect conventional threshold-based detection systems. Engagement with the WADA Science Department and prohibited list scientific committee regarding the regulatory classification of nanotechnological supplementation vehicles is strongly recommended.

#### 4.4.3. Regulatory Status of Nanotechnology-Based Medical Devices

Taopatch^®^ holds CE Class I medical device marking under EU MDR 2017/745. Class I devices are those with the lowest risk classification, for which manufacturers may self-certify conformity with general safety and performance requirements without independent notified body review of clinical evidence (with certain exceptions). This classification does not imply demonstration of clinical efficacy by any regulatory authority. Claims of performance enhancement in elite athletes exceeding the device’s CE marking scope require independent scientific scrutiny and should not be presented as established fact in scientific publications or promotional materials.

#### 4.4.4. Note on WADA and Regulatory Statements

The discussion of WADA anti-doping regulations and EMA/AIFA regulatory frameworks in Section 4.4.2 and Section 4.4.3 is provided as contextual information relevant to the practical and regulatory environment surrounding nanotechnological applications in sport. These statements derive from publicly available regulatory and legal documents, not from the systematic search conducted for this review. They should not be interpreted as part of the primary evidence synthesis and are clearly distinguished from the evidence map findings. Athletes, physicians, and researchers should consult directly with the relevant authorities (WADA Science Department; EMA; national regulatory agencies) for authoritative regulatory guidance, as regulatory classifications may be updated after the search date of this review.

### 4.5. Limitations of This Systematic Scoping Review

This systematic scoping review has several limitations, each of which is acknowledged explicitly. First, the search was limited to PubMed/MEDLINE, Scopus, and Web of Science; SPORTDiscus, Embase, IEEE Xplore, and discipline-specific engineering and materials science databases were not searched, introducing retrieval bias with differential impact across domains (largest for wearable sensors and materials engineering). Second, grey literature, trial registries, and non-English publications were not systematically searched, introducing publication and language bias; the volume of nanotechnology research published in Chinese-language journals is acknowledged as a particular source of language bias. Third, the review was not prospectively registered on PROSPERO or OSF; post hoc modification of eligibility criteria cannot be externally excluded, and readers should interpret findings with proportionate caution. Fourth, meta-analysis and formal GRADE assessment were not feasible due to substantial heterogeneity across all dimensions. Fifth, inter-rater agreement was not prospectively quantified; Cohen’s κ and percentage agreement were not calculated or formally recorded. Sixth, several included domains relied predominantly on animal, technical, materials-science, or narrative-review evidence rather than direct athlete trials. Seventh, standardized quality appraisal tools were not available for all evidence types, requiring narrative appraisal for materials-science studies and narrative reviews, which are less validated. Eighth, 16 of the 28 included records are secondary or contextual sources (Stratum 4) that may indirectly cite overlapping primary literature, limiting the interpretability of the 28-record evidence map as 28 independent evidence bodies. Ninth, the absence of PRISMA-ScR prospective registration and the use of PRISMA 2020 in an earlier draft submission are acknowledged methodological limitations now corrected.

### 4.6. Future Research Directions

Priority areas for future investigation include:

- Adequately powered, double-blind, placebo-controlled RCTs of nano-antioxidant supplementation in elite human athletes, with pre-specified outcomes and prospective PROSPERO registration.
- Independent nanomaterial characterization of commercial PBM nanotechnology devices (Taopatch^®^ and analogues) via peer-reviewed spectroscopic analysis, including measurement of photon emission spectra and irradiance delivered to target tissues.
- Formal pharmacokinetic and pharmacodynamic studies of nano-encapsulated antioxidants (resveratrol-SLN, CoQ10-NP, ROS-scavenging NPs) in human volunteers, establishing safety, bioavailability, and dose–response data as prerequisites to elite athlete trials.
- Development of QD-based wearable sensor systems validated in real competition environments, prioritizing heavy-metal-free formulations.
- Long-term safety studies of CNT-containing materials in regular contact with athletes, with full product lifecycle assessment from manufacturing to disposal.
- Standardization of performance outcome measurement using validated, sport-specific metrics to enable future meta-analyses.
- Prospective registration of all future systematic reviews in this domain on PROSPERO and all clinical trials on ClinicalTrials.gov.
- Engagement with WADA and EMA to establish regulatory frameworks for nanotechnological supplementation vehicles and nano-enabled medical devices in sport.
- Inclusion of grey literature and clinical trial registry searching in future iterations of this review to reduce publication bias.
- Application of GRADE certainty-of-evidence ratings per outcome domain in future reviews with sufficient primary study evidence.
- Formal reporting of inter-rater agreement (Cohen’s κ with 95% CI) at both screening stages in all future iterations.

## 5. Conclusions

Nanotechnology represents a theoretically promising but currently immature frontier in sports science. Applying the three-stage interpretive framework used throughout this review:

- At the technological feasibility stage, CNT-reinforced materials and wearable nanosensor platforms are the most technically advanced domains, with reproducible materials-science validation. At the physiological/biomechanical validation stage, limited human evidence exists for CNT-based insoles (one proof-of-concept crossover study in healthy non-elite males) and CNT motion sensors (one human validation study). At the demonstrated athletic performance benefit stage, no domain has sufficient high-quality direct evidence to support routine application for elite athletic performance enhancement.
- Nano-antioxidant strategies show consistent effects in animal exercise models (Stratum 3), but no controlled human athlete data exist; the translational gap remains entirely unbridged. Conventional PBMT systematic reviews establish the biological plausibility of photobiomodulation as a device category with moderate-confidence evidence from multiple RCTs; however, evidence from conventional PBMT devices cannot be extrapolated to Taopatch^®^ or any nanotechnology-enabled PBM device without independent spectroscopic characterization and dosimetric verification, which does not currently exist for Taopatch^®^ in the peer-reviewed literature. QD-based applications remain largely in the theoretical stage for elite sport, though heavy-metal-free QD photodetectors and GQD biosensors represent a technically feasible future direction for wearable monitoring.
- Future studies across all domains must be prospectively registered on PROSPERO and ClinicalTrials.gov, adequately powered, independently characterized, conducted in clearly defined trained or elite athlete populations, and use standardized, sport-specific outcomes with appropriate regulatory and anti-doping oversight.

## Figures and Tables

**Table 1:** Applied within the Population–Concept–Context (PCC) scoping review framework. Stratum 4 has been subdivided into Stratum 4a (review-type studies) and Stratum 4b (materials science studies) to reflect substantively different methodological standards, quality appraisal tools, and interpretive weight applicable to each subgroup. TOTAL INCLUDED RECORDS: 28 (Stratum 1: n = 2; Stratum 2: n = 7; Stratum 3: n = 3; Stratum 4a subgroup i: n = 5; Stratum 4a subgroup ii: n = 7; Stratum 4b: n = 4). Notes on Stratum Boundaries and Classification Decisions, Stratum 1 population criterion: Stratum 1 classification is determined exclusively by study design (randomized or quasi-randomized controlled trial; controlled crossover trial; controlled before-after study with a concurrent control group) and is not conditional on the training status of the enrolled population. The training status of enrolled participants (elite athlete, trained athlete, or healthy/non-elite subject) is extracted and reported in the data extraction table (Supplementary Material Section S4) as a separately classified characteristic that qualifies the directness and generalizability of the evidence, but does not constitute an eligibility criterion for stratum assignment. Studies enrolling non-elite or healthy participants that satisfy the controlled-design criterion are classified at the lower boundary of Stratum 1 and are explicitly described as proof-of-concept controlled human evidence—contributing to the evidence map as Stratum 1 entries without implying equivalence with controlled trials conducted in trained or elite athlete populations. Both Stratum 1 records included in this review (Ko et al. [17]; Malchiodi Albedi et al. [11]) enrolled healthy non-athlete populations and are therefore consistently described as lower-boundary Stratum 1 evidence throughout the manuscript.

Stratum	Stratum Descriptor	Eligible Study Designs	Population/Sample Requirements	Required Nanotechnological Intervention	Comparator Requirements	Minimum Outcome Requirement	Quality Appraisal Tool	Included Studies (n)
Stratum 1	Direct Controlled Human Evidence	Randomized controlled trials (RCTs); controlled crossover trials (randomized or quasi-randomized); controlled before-after studies with a concurrent control group	Human participants of any age, sex, or training status (including healthy volunteers, recreational athletes, trained athletes, and elite athletes); no restriction on clinical condition if a sport- or exercise-relevant performance outcome is measured	Any nanotechnological material or device applied to or administered in a human participant with the direct aim of modifying athletic or exercise performance, recovery, monitoring capacity, or biomechanical properties relevant to sport; includes externally applied devices (e.g., nanosensor arrays, nano-composite equipment, photoselective nanomaterial patches), orally administered nano-encapsulated compounds, and wearable nanomaterial-based sensors used in a human exercise context	An active comparator, sham/placebo condition, or standard-of-care control group; pre-post crossover designs accepted where each participant serves as their own control with adequate washout documentation	At least one quantitative outcome in any of the following domains: physical performance (e.g., time to exhaustion, jump height, sprint time, VO_2_max, peak power output); biomechanical performance (e.g., joint kinematics, force output, balance/postural control); physiological markers of performance or recovery (e.g., blood lactate, creatine kinase, oxidative stress markers, heart rate); wearable sensor performance validated against a reference standard in a human exercise context	Cochrane Risk of Bias 2 (RoB 2) tool [18]	2
Stratum 2	Observational and Quasi-Experimental Human Evidence	Prospective cohort studies; retrospective cohort studies; cross-sectional studies; pre-post studies without a concurrent control group (single-arm); case series (minimum n = 5); sensor validation studies with a human participant cohort; quasi-experimental studies with non-randomized allocation	Human participants of any age, sex, or training status; includes non-athlete and clinical populations where a sport- or exercise-relevant performance outcome (physical performance, balance/postural control, exercise capacity, physiological monitoring) is measured and directly relevant to the scoping review concept	As per Stratum 1	A pre-intervention baseline measurement; an alternative nanomaterial configuration (for sensor validation studies); or a concurrent non-intervention group (if available); a concurrent comparator group is desirable but not required for stratum eligibility	At least one quantitative outcome in any of the performance, physiological, biomechanical, or wearable sensor performance domains defined under Stratum 1	Newcastle–Ottawa Scale (NOS) for cohort and case–control studies [19]	7
Stratum 3	Translational Animal Exercise Models (restricted a priori to the nano-supplementation/nano-antioxidant domain)	Controlled in vivo animal experiments using validated exercise or exhaustion models (treadmill, swim-to-exhaustion, wheel running, or equivalent); animal models only; in vitro studies excluded from this stratum	Non-human animal subjects (rodent models preferred, as most established in exercise physiology research); no restriction on species, sex, or age of animal	Nano-encapsulated or nanoparticle-formulated nutritional or pharmacological compounds (e.g., solid lipid nanoparticles, reactive nanoparticle antioxidants, nanostructured lipid carriers) administered by any route; the nanomaterial formulation must be directly compared to either a free (non-nano) equivalent compound or a vehicle control	A free (non-nano) equivalent compound at an equivalent or comparable dose; a vehicle control; or a sham administration condition	At least one quantitative exercise performance outcome (e.g., time to exhaustion, distance run, swim time); and/or at least one mechanistically relevant biochemical outcome (e.g., oxidative stress markers, antioxidant enzyme activity, tissue integrity markers) with direct relevance to the hypothesized mechanism of performance enhancement	SYRCLE Risk of Bias Tool for animal studies [20]	3
Stratum 4a	Review-Type Studies: True Systematic Reviews, Meta-Analyses, and Narrative/Comprehensive Reviews ‡	Subgroup (i)—True systematic reviews and meta-analyses: Studies explicitly reporting a pre-defined search strategy, eligibility criteria, and quantitative or qualitative evidence synthesis; Cochrane reviews; PRISMA-compliant systematic reviews with or without meta-analysis	Subgroup (i): Any human population with outcomes in the performance, physiological, biomechanical, or wearable monitoring domains; in the PBM sub-domain, systematic reviews of photobiomodulation therapy (PBMT/LLLT/LED) in human athletes or exercise populations are included as contextual PBM framework evidence even when not specific to nanotechnological devices (see Section 2.2 and Supplementary Material Section S2 for justification)	Subgroup (i): Any nanotechnological intervention as defined for Strata 1–2; OR, in the PBM sub-domain, any characterised PBMT device (including conventional LLLT and LED systems) evaluated as contextual PBM framework evidence	Not required as an eligibility criterion for review-type studies; evaluated as reported in each included review	A synthesized quantitative or qualitative conclusion on at least one performance, physiological, biomechanical, or monitoring outcome domain relevant to the scoping review PCC framework	Subgroup (i): AMSTAR-2 [21]; rated as high, moderate, low, or critically low confidence	5 (subgroup i)
Subgroup (ii)—Narrative and comprehensive reviews: Reviews explicitly designated as narrative, comprehensive, or scoping reviews; reviews synthesizing technology-enabling or mechanistic evidence across multiple primary studies without formal systematic search methodology	Subgroup (ii): Any study population relevant to the sport/exercise nanotechnology domain; includes basic science, engineering, and clinical population reviews where the synthesis is directly relevant to one of the four primary domains (CNT-based equipment, nano-supplementation, wearable nanosensors, QD-based monitoring, or PBM nanotechnology)	Subgroup (ii): Any nanotechnological material or device as defined for Strata 1–2; OR foundational characterisation of nanomaterial properties (e.g., QD photophysics, CNT mechanical properties, graphene electrochemistry) with direct, explicitly articulated relevance to sport or exercise applications	Not required	A synthesized conclusion on nanomaterial properties, clinical/performance implications, or translational potential relevant to the scoping review PCC framework	Subgroup (ii): Locally developed Structured Narrative Quality Appraisal Framework (6-domain; Domains A–F)—see Supplementary Material Section S5.1; rated as Methodologically Transparent/Partial Transparency/Limited Transparency	7 (subgroup ii)		
Stratum 4b	Materials Science Studies	Laboratory materials characterisation studies; computational modelling studies (finite element analysis, molecular dynamics simulation); proof-of-concept engineering studies with or without a small human demonstration component (PoC); materials synthesis and testing studies	Primary model: Laboratory specimens, test pieces, or computational representations of sports equipment materials, functional textiles, or nanosensor device components; Human component (if present): Healthy volunteers used for proof-of-concept device demonstration only (not a formal clinical cohort); formal human participant outcomes from a PoC component are reported descriptively only and do not contribute to Stratum 1–2 evidence	Any nanomaterial directly relevant to sport equipment, functional athletic textiles, or wearable sensor device fabrication, including CNTs (SWCNT, MWCNT), graphene, graphene quantum dots, nanocomposite polymer matrices, and allied nanomaterial systems; the sport or athlete performance application must be explicitly stated by the study authors	Not required for materials science studies; alternative nanomaterial configurations used as internal comparators are described where present	At least one quantitative mechanical, electrochemical, optical, or electromechanical performance metric directly relevant to the stated sport or athlete application (e.g., Young’s modulus, tensile strength, wear resistance, strain range, gauge factor, detection limit, signal-to-noise ratio, stiffness-to-weight ratio)	Locally developed Structured Materials Science Quality Appraisal Framework (6-domain; Domains I–VI)—see Supplementary Material Section S5.2; rated as High/Moderate/Limited-to-Moderate Methodological Rigour	4

**Table 2:** Eligibility Exclusion Criteria Applied Across All Strata.

Exclusion Criterion	Rationale
In vitro studies (cell culture, acellular systems) not accompanied by an in vivo or human validation component	Insufficient translational proximity to the athlete performance context; below the minimum evidence stratum threshold
Studies in which the nanotechnological component could not be clearly identified and distinguished from other co-interventions	Nanotechnological contribution to any observed outcome is unevaluable
Conference abstracts, letters, editorials, and opinion pieces without original primary or synthesis data	Insufficient reportable data for extraction; not peer-reviewed as full manuscripts
Studies for which full text was unavailable after two independent retrieval attempts (institutional access + direct author contact)	Full data extraction not possible
Studies not published in English, Italian, French, Spanish, or Portuguese	Language competence of the review team limited to these five languages; applies to full texts only (abstracts in any language screened at title/abstract stage)
Studies evaluating nanotechnological interventions exclusively in non-exercise clinical or pharmacological contexts with no direct or inferential relevance to sport or exercise performance	Outside the defined Concept boundary of the PCC framework

**Table 3:** Characteristics of Included Studies. Abbreviations: CNT = carbon nanotube; QD = quantum dot; RCT = randomized controlled trial; SLN = solid lipid nanoparticle; RNPN = pH-sensitive self-assembling reactive nanoparticle (subcutaneous); RNPO = ROS-scavenging nanoparticle (oral, gastrointestinal-targeted); PBM = photobiomodulation; PBMT = photobiomodulation therapy; RoB = risk of bias; SR = systematic review; MA = meta-analysis; GI = gastrointestinal; RBC = red blood cells; MS = multiple sclerosis; PPG = photoplethysmography; CFI = carbon fiber insole; CoQ10 = coenzyme Q10; SWCNT = single-walled carbon nanotube; MWCNT = multi-walled carbon nanotube; ISE = ion-selective electrode; NOS = Newcastle–Ottawa Scale; SYRCLE = Systematic Review Centre for Laboratory animal Experimentation; GQD = graphene quantum dot; NR = not reported in source publication; D = domain (RoB 2); SOD = superoxide dismutase; MDA = malondialdehyde; LPS = lipopolysaccharide; CoI = conflict of interest; HR = heart rate; SpO_2_ = peripheral oxygen saturation; ↑ = improve; ↓ = decrease. ★ = quality star as assigned by the Newcastle–Ottawa Scale (NOS) [19]; scores range from 0 to 9 stars across three domains (Selection, Comparability, and Outcome/Exposure); studies scoring ≤4★ are classified as high risk of bias; 5–6★ as moderate risk of bias; ≥7★ as low risk of bias. NOS was applied exclusively to Stratum 2 observational and quasi-experimental studies; RoB 2 was applied to Stratum 1 controlled trials; SYRCLE was applied to Stratum 3 animal studies; AMSTAR-2 was applied to Stratum 4a true systematic reviews and meta-analyses. ★ symbol is not used for RoB 2, SYRCLE, or AMSTAR-2 quality appraisal results in this table.

STRATUM 1—DIRECT RCT/CONTROLLED TRIAL EVIDENCE (n = 2)											
#	First Author, Year [Ref]	Country	Domain	Study Design	Population/n	Nanotechnological Intervention	Comparator	Primary Outcome(s)	Key Finding(s)	QA Tool	QA Result
1	Ko, 2023 [17]	Republic of Korea	CNT-based sports equipment	Controlled within-subject crossover trial	Healthy adult males/n = 30	Carbon fibre insole (CFI) incorporating CNT-reinforced composite	Conventional standard insole	Vertical jump height; agility; sprint speed	↑ Vertical jump +1.5% (*p* < 0.05); ↑ agility performance (*p* < 0.05); no statistically significant sprint effect; sample size calculation and MCID threshold NR	RoB 2	Some concerns overall—D4: outcome assessor blinding insufficiently described; D5: no pre-registered protocol; selective reporting not excludable
2	Malchiodi Albedi, 2018 [11]	Italy	PBM nanotechnology (Taopatch^®^)	Parallel-group RCT	Healthy subjects/n = 40	Taopatch^®^ (CE Class I photoselective nanomaterial patch, body-heat-activated, claimed 400–1400 nm emission)	Sham device	Postural control parameters (stabilometry)	↑ Postural control vs. sham (*p* < 0.05); nanomaterial composition and emission dose not independently characterised; authors qualified as preliminary data; non-athlete population	RoB 2	High risk overall—D1: randomisation process not fully described; D2: sham blinding feasibility not documented; D4: outcome assessor blinding insufficient; D5: no pre-registered protocol; selective reporting not excludable
STRATUM 2—OBSERVATIONAL-EXPERIMENTAL HUMAN EVIDENCE (n = 7)											
#	First Author, Year [Ref]	Country	Domain	Study Design	Population/n	Nanotechnological Intervention	Comparator	Primary Outcome(s)	Key Finding(s)	QA Tool	QA Result
3	Messina, 2022 [12]	Italy	PBM nanotechnology (Taopatch^®^)	Prospective observational	MS patients/n = 20 (8M, 12F)	Taopatch^®^ + structured proprioceptive rehabilitation protocol	No separate control group	Baropodometric balance; bilateral handgrip strength; serum 25-OH vitamin D	↑ Postural balance (*p* < 0.05); ↑ handgrip strength bilaterally (right: *p* = 0.023; left: *p* = 0.021); trend to ↑ vitamin D, NS; non-athlete population; mechanism unexplained	NOS	4★/9—High RoB
4	Carbonari, 2021 [22]	Italy	PBM nanotechnology (Taopatch^®^)	Retrospective cross-over study	Competitive athletes/n = 25	Taopatch^®^ device	Occlusal splint	Muscular performance indices; postural balance; posture parameters	Both interventions ↑ measured parameters; absence of true sham control—placebo effect not excludable; retrospective unblinded design	NOS	4★/9—High RoB
5	Amato, 2021 [23]	Italy	PBM nanotechnology (Taopatch^®^)	Pilot pre-post study (non-RCT)	MS patients during COVID-19 lockdown/n = 10	Taopatch^®^ + home-based exercise training protocol	Pre-intervention baseline only	Sedentary behaviour (daily activity); biochemical markers	↓ Sedentary time; changes in biochemical markers; combined intervention—effects of Taopatch^®^ not separable from exercise component; non-athlete population	NOS	3★/9—Likely high RoB
6	Wyckoff, 2025 [24]	USA	Wearable nanosensors	Prospective sensor validation study (human)	n = 20 (n = 10 per material group)	MWCNT elastic fabric motion tape sensors	Graphene nanosheet tape sensors	Low back movement characterisation accuracy (forward flexion, lateral bending, axial rotation)	MWCNT sensors accurately characterised all low back movement types; MWCNT > graphene nanosheet for signal stability under high-strain conditions; validated for sports biomechanics applications	NOS	8★/9—High quality
7	Campoli, 2026 [25]	Italy	PBM nanotechnology (Taopatch^®^)	Pilot quasi-experimental study	Hearing aid users/n = 40 (19M, 21F; mean age 77.3 ± 15.6 y)	Taopatch^®^ photoselective nanomaterial patch integrated into hearing aid	Hearing aid alone (standard condition)	Posturographic balance parameters	Preliminary ↑ balance parameters; multimodal intervention structure precludes attribution to nanotechnological component; non-athlete, elderly population	NOS	3★/9—Likely high RoB
8	Akiyama, 2022 [26]	Japan	Externally applied nanomaterial (IFMC)	Acute treadmill exercise study	Athletic club students at Fukushima University/n = 18 (106 total measurements)	Externally applied natural-mineral-based novel nanomaterial IFMC (Japanese hot spring-derived; haematite, olivine, rhodolite, zincite)	Sham/control condition	HR during aerobic exercise; VO_2_; VO_2_max; CO_2_ production; respiratory quotient	↓ HR elevation in IFMC group (*p* < 0.05); no significant difference in VO_2_, VO_2_max, CO_2_, or RQ; preliminary evidence of cardiovascular function improvement; precise mechanism under investigation	NOS	4★/9—Moderate
9	Li S, 2023 [27]	China/USA	QD-based wearable biosensing	Engineering + human validation study	n = 5	Cu-In-Se quantum dot wearable PPG photodetector (heavy-metal-free formulation)	Conventional reference monitoring device	HR fidelity; SpO_2_ monitoring accuracy; mechanical flexibility of device	High-fidelity PPG signal; mechanical flexibility adequate for wearable athlete monitoring application; heavy-metal-free formulation addresses cytotoxicity barrier of conventional CdSe/CdTe QDs	NOS	5★/9—Moderate quality
STRATUM 3—TRANSLATIONAL ANIMAL EXERCISE MODELS (Nano-antioxidant domain only; n = 3)											
#	First Author, Year [Ref]	Country	Domain	Study Design	Animal Model/n	Nanotechnological Intervention	Comparator	Primary Outcome(s)	Key Finding(s)	QA Tool	QA Result
10	Toriumi, 2021 [6]	Japan	Nano-antioxidant supplementation	In vivo animal exercise experiment	Wistar rats (male)/n ≈ 7 per group	RNPN: pH-sensitive self-assembling antioxidant nanoparticle (subcutaneous administration)	Vehicle control	Treadmill running time to exhaustion (40 m/min); post-exercise circulating RBC count	↑ Running time ~50% (60 → 90 min; *p* < 0.05); ↓ post-exercise RBC loss (protective against exercise-induced oxidative haemolysis); subcutaneous route—no clinically equivalent athlete administration route	SYRCLE	Unclear risk—sequence generation, allocation concealment, and outcome assessor blinding inadequately reported
11	Toriumi, 2023 [7]	Japan	Nano-antioxidant supplementation	In vivo animal exercise experiment	C57BL/6J mice (male)/n = 7–8 per group	RNPO: oral GI-targeted ROS-scavenging antioxidant nanoparticle (dose-escalation design)	Free antioxidant TEMPOL (oral); vehicle control	Running time to exhaustion; GI mucosal integrity; plasma LPS concentration	↑ Running time (dose-dependent; *p* < 0.05); ↓ plasma LPS; GI mucosa protected vs. vehicle; TEMPOL ↓ running time at high dose—nano-formulation superior to conventional antioxidant at equivalent dose	SYRCLE	Unclear risk—multiple domains insufficiently reported
12	Qin, 2020 [8]	China	Nano-antioxidant supplementation	In vivo animal exercise experiment	ICR mice (male)/n = 10 per group	RSV-SLN: resveratrol-loaded solid lipid nanoparticle (oral)	Free resveratrol (oral, equivalent dose); vehicle control	Exhaustive running time; SOD activity; MDA concentration; SIRT1/autophagy pathway markers (LC3-II, p62)	↑ Running time vs. free RSV (*p* < 0.05); ↑ SOD; ↓ MDA; SIRT1/autophagy pathway identified as mechanistic mediator; nano-encapsulation overcomes poor oral bioavailability of free RSV	SYRCLE	Unclear risk—multiple domains insufficiently reported
STRATUM 4a—SYSTEMATIC REVIEWS AND META-ANALYSES (n = 5)											
#	First Author, Year [Ref]	Country	Domain	Study Design	Included Studies/n	Nanotechnological Intervention Category		Primary Outcome(s)	Key Finding(s)	QA Tool	QA Result
13	Leal-Junior, 2015 [28]	Brazil/International	PBM—exercise performance (contextual framework)	SR + MA (PEDro ≥ 6/10 quality threshold)	16 RCTs retrieved; 13 included in analysis (≥6/10 PEDro); participants NR per included study	Phototherapy (LLLT + LED; pre-, during, and post-exercise)		Number of repetitions to failure; time to exhaustion; CK activity	↑ Time to exhaustion (MD +4.12 s; 95% CI 1.21–7.02; *p* < 0.005); ↑ repetitions (MD +5.47; 95% CI 2.35–8.59; *p* < 0.0006); heterogeneity precluded biochemical MA; most consistent results: red/infrared; pre-exercise application; 50–200 mW; 5–6 J/point	AMSTAR-2	Moderate confidence
14	Vanin, 2018 [29]	Brazil/International	PBM—muscular performance (contextual framework)	SR + MA	39 RCTs included (861 participants total); 28 RCTs included in MA	PBMT (LLLT and/or LED; pre-exercise and exercise-concomitant; 655–950 nm range)		Time to exhaustion; number of repetitions; isometric peak torque; blood lactate	↑ Muscular performance (time to exhaustion, repetitions, torque); ↓ fatigue-related lactate; largest effect: immediate pre-exercise application; 20–60 J (small muscles), 60–300 J (large muscles); very low to moderate quality of evidence	AMSTAR-2	Moderate confidence
15	Leal-Junior, 2019 [30]	Brazil/International	PBM—clinical dosimetry recommendations (contextual framework)	SR + clinical recommendations	Post-2018 RCTs + prior SR/MA data (n of included primary studies NR in abstract)	PBMT (660–905 nm; recommended 20–50 J/muscle group pre-exercise)		Exercise performance; post-exercise recovery	Pre-exercise PBMT at optimal dosimetric parameters (wavelength 660–905 nm; 20–50 J/muscle) = most consistent ergogenic results; dosimetry identified as primary determinant of efficacy; further high-quality RCTs required	AMSTAR-2	Low confidence—narrative recommendation component; protocol registration status unclear
16	Dutra, 2022 [31]	Brazil/International	PBM—mode-specific exercise performance (contextual framework)	SR + MA	37 individual studies; 78 exercise performance measurements; 586 participants	PBMT (characterised devices with defined dosimetric parameters)		Mode-specific exercise performance: muscle endurance; cycling time to exhaustion; muscle strength; sprint; running; swimming	↑ Muscle endurance (SMD 0.27; 95% CI 0.12–0.41; *p* < 0.01); ↑ cycling time to exhaustion (SMD 0.35; 95% CI 0.10–0.59; *p* < 0.01); no significant effect on muscle strength, running, swimming performance; mode-specific and parameter-dependent effects	AMSTAR-2	Moderate confidence
17	De Marchi, 2022 [32]	Brazil/International	PBM—exercise-induced oxidative stress (contextual framework)	SR + MA	n of included RCTs NR in abstract; healthy humans	PBMT (characterised devices; pre-exercise and post-exercise)		Exercise-induced lipid peroxidation; protein carbonylation; SOD activity; catalase activity	↓ Lipid peroxidation; ↓ protein carbonylation; ↑ SOD activity; ↑ catalase activity; PBMT attenuates exercise-induced oxidative stress and enhances endogenous antioxidant enzyme response	AMSTAR-2	Moderate confidence
STRATUM 4a—NARRATIVE/COMPREHENSIVE REVIEWS (n = 7)											
#	First Author, Year [Ref]	Country	Domain	Study Design	Review Scope	Nanotechnological Intervention Category		Key Topic(s)	Key Finding(s)	QA Tool	QA Result
18	Pisanic, 2014 [14]	USA	QD-based biosensing	Narrative review	In vitro and preclinical diagnostics	QDs for diagnostics and detection (multiple formulations)		Detection sensitivity; photostability; multiplexing	Superior sensitivity, photostability, and multiplexing capacity vs. organic fluorophores; foundational QD biosensing characterisation	Structured narrative quality framework	Clear question; selective citation pattern; search strategy NR; CoI NR
19	Seshadri, 2019 [9]	USA	Wearable nanosensors	Narrative review	Athlete workload monitoring technologies	Nano-electrochemical sweat sensors for real-time physiology		Internal/external athlete workload monitoring	Nanomaterial-based electrochemical sensors identified as most promising emerging technology for non-invasive real-time athlete physiological monitoring (lactate, glucose, electrolytes)	Structured narrative quality framework	Clear question; search partially described; no formal eligibility criteria; CoI NR
20	Pirzada, 2019 [10]	UK/Turkey	Wearable nanosensors	Comprehensive review	Healthcare and sport biosensing	CNT/graphene-based transducers and biosensor platforms		Detection limit; metabolite selectivity	Orders-of-magnitude lower detection limit vs. conventional electrode materials; high selectivity for exercise-relevant metabolites; direct sport applicability confirmed	Structured narrative quality framework	Clear question; broad coverage; no systematic search; CoI declared
21	Wagner, 2019 [13]	USA	QD-based biosensing	Comprehensive review	Biomedical and preclinical QD applications	Multiple QD formulations (CdSe, CdTe, InP, carbon QDs, GQDs)		Biomedical applicability; in vivo feasibility; cytotoxicity profile	>10,000 publications in QD biomedical applications; CdSe/CdTe cytotoxicity and limited in vivo clearance data = primary clinical translation barriers; heavy-metal-free alternatives in development	Structured narrative quality framework	Clear question; broad coverage; no systematic search; CoI declared
22	Li MY, 2025 [2]	China	Cross-domain—nanotechnology in sport	Comprehensive review	Multiple sport domains	Nanotechnology applications in sport (broad overview)		Wearable sensors; functional fabrics; sports medicine; equipment engineering	Wearable sensors, functional fabrics, and sports medicine identified as the most technologically developed nanotechnology-in-sport application domains	Structured narrative quality framework	Clear question; broad domain coverage; no systematic search; CoI declared
23	Fathy, 2025 [33]	USA/International	Wearable nanosensors	Comprehensive review	Flexible and wearable potentiometric sensor platforms	CNT-based solid-contact ISEs; nanoporous carbon transducers for multi-analyte sweat monitoring		Multi-analyte sweat monitoring (Na^+^, K^+^, pH, NH_4_^+^, lactate)	Athlete sweat analysis confirmed as primary application domain; exercise-validated potentiometric CNT systems demonstrated; real-time multi-analyte monitoring feasible in exercise contexts	Structured narrative quality framework	Clear question; search partially described; broad evidence coverage; CoI NR
24	Tiwari, 2021 [34]	India	QD-based biosensing	Comprehensive review	Healthcare and preclinical QD applications (broad)	QD formulations in healthcare (diagnostics, drug delivery, imaging)		Healthcare advancement potential; translational feasibility	Wide potential applicability across healthcare and diagnostics; cytotoxicity and regulatory compliance = primary barriers to clinical translation	Structured narrative quality framework	Clear question; broad coverage; search methods NR; CoI declared
STRATUM 4b—MATERIALS SCIENCE STUDIES (n = 4)											
#	First Author, Year [Ref]	Country	Domain	Study Design	Study Model	Nanotechnological Intervention/Material		Outcome Measure(s)	Key Finding(s)	QA Tool	QA Result
25	Harifi, 2017 [3]	Iran	CNT-based sports equipment and textiles	Narrative review of materials science evidence	Lab specimens and published materials data	CNT-reinforced sports racquet frames and functional textiles		Deformation resistance; frame stiffness; functional textile properties	~50% ↑ deformation resistance vs. standard carbon racquets; CNT integration improves both structural and functional textile properties for sport	Narrative appraisal	Broad materials coverage; no formal systematic methodology; no independent mechanical testing reported
26	Nurazzi, 2021 [4]	Malaysia	CNT-based sports equipment	SR (materials science focus)	Lab specimens (polymer composite test pieces)	CNT-reinforced polymer composites (varied CNT loading fractions)		Young’s modulus; tensile strength; wear resistance (block-on-ring testing)	Consistent ↑ Young’s modulus and tensile strength with ↑ CNT content; +50% wear resistance vs. neat polymer matrix; data reproducible across multiple composite configurations	Narrative appraisal	Quantitative materials data; validated mechanical testing protocols; no clinical PICOS applied; sport-performance extrapolation inferential
27	Miao, 2023 [5]	China	CNT-based sports equipment	Materials science + computational study	Lab specimens and finite element/computational models	CNT/graphene composite configurations (optimised processing parameters)		Stiffness-to-weight ratio; energy transfer efficiency; lightweight–strength trade-off index	Significant optimisation of lightweight–strength trade-off achieved; improved bicycle frame stiffness-to-weight ratio and energy transfer efficiency vs. conventional carbon composites	Narrative appraisal	Quantitative materials and computational data; limited clinical translation discussion; performance extrapolation to elite athletes is inferential
28	Yamada, 2011 [35]	Japan	Wearable nanosensors	Materials science characterisation + PoC human demonstration	Lab characterisation + individual human volunteers (PoC demonstration; no formal clinical cohort; sample size NR)	SWCNT stretchable strain sensor (CNT/PDMS composite, aligned array)		Strain range (% elongation at failure); gauge factor; human motion detection fidelity (joint movement, breathing, speech)	Up to 280% strain (approximately 50× conventional metal strain gauges); high gauge factor; successfully detected full range of complex human movements, breathing patterns, and speech; landmark nanosensor characterisation study	Narrative appraisal	Landmark materials science publication; gold-standard SWCNT sensor characterisation; human component = PoC demonstration only; formal sample size for human validation not reported as clinical cohort

**Table 4:** Domain-stratum evidence summary matrix. Abbreviations: CNT = carbon nanotube; PoC = proof of concept; PBM = photobiomodulation; PBMT = photobiomodulation therapy; QD = quantum dot; PPG = photoplethysmography; SR/MA = systematic review/meta-analysis; RoB = risk of bias.

Domain	Technological Feasibility (Stratum 4)	Physiological/Biomechanical Validation in Humans (Strata 1–2; Lower-Boundary Stratum 1 Studies in Non-Elite Populations Placed Here)	Direct Athletic Performance Evidence (Target: Stratum 1, Trained/Elite Population; Absent in All Domains)	Aggregate Risk of Bias (Strata 1–2)	Consistency of Findings
CNT-based equipment	Established (materials science)	Limited PoC (1 crossover, n = 30, non-elite)	Absent	Some concerns	Consistent across materials; single human study
Nano-antioxidant supplementation	Established (animal models only)	Absent	Absent	Unclear (animal studies only)	Consistent across 3 rodent models
Wearable nanosensors	Established (materials + engineering)	Partial (1 validation study, n = 20)	Absent	Low–Moderate	Consistent in technical; limited human data
PBM—conventional PBMT (contextual)	N/A—not a nanotechnological intervention	Established (multiple RCTs)	Moderate evidence in non-elite populations	Low–Moderate (SR/MA level)	Consistent for specific dosimetric parameters
PBM—Taopatch^®^ (nano-enabled)	Not independently characterized	Preliminary, high-RoB studies in non-athletes	Absent	High	Inconsistent; multiple confounders
QD-based biosensing	Partial (technical proof-of-concept)	Very limited (n = 5, PPG validation)	Absent	Moderate (single study)	Insufficient data for assessment

Footnotes. The column headers reflect the three-stage translational evidence progression framework applied throughout Section 3.3 and Section 4.1 (technological feasibility → physiological/biomechanical validation in humans → demonstrated athletic performance benefit in trained/elite athletes), and do not represent formal stratum eligibility boundaries. The ‘Physiological/Biomechanical Validation in Humans’ column encompasses evidence from Stratum 2 observational studies and from lower-boundary Stratum 1 controlled studies in which the enrolled population comprised non-elite or healthy subjects (specifically: Ko et al. [18] in the CNT-based equipment domain; Malchiodi Albedi et al. [11] and Stratum 2 studies in the PBM, Taopatch^®^ domain; Li et al. 2023 [27] in the QD biosensing domain). The ‘Direct Athletic Performance Evidence in Trained/Elite Athletes’ column reflects the current evidence gap: no adequately powered controlled study enrolling trained or elite athletes has been conducted in any nanotechnological sub-domain reviewed here.

## Data Availability

All data supporting the findings of this systematic review are available within the article. The complete search strategies, inclusion and exclusion criteria, and data extraction templates are available from the corresponding author upon reasonable request.

## Supplementary Materials

The following supporting information can be downloaded at https://www.mdpi.com/article/10.3390/jfmk11040416/s1, Section S1: Search strategies; Section S2: List of Contextual References; Section S3: Preferred Reporting Items for Systematic reviews and Meta-Analyses extension for Scoping Reviews (PRISMA-ScR) Checklist; Section S4: Full Data Extraction Table; Section S5: Structured Narrative Quality Appraisal Criteria [51,52].

## Abbreviations

AMSTAR-2	A MeaSurement Tool to Assess systematic Reviews, version 2
CFI	Carbon fibre insole
CNT	Carbon nanotube
CoQ10	Coenzyme Q10
EIGS	Exercise-induced gastrointestinal syndrome
EMA	European Medicines Agency
EMG	Electromyography
GI	Gastrointestinal
GRADE	Grading of Recommendations Assessment, Development and Evaluation
GQD	Graphene quantum dot
HR	Heart rate
ISE	Ion-selective electrode
LPS	Lipopolysaccharide
LLLT	Low-level laser therapy
MA	Meta-analysis
MDA	Malondialdehyde
MeSH	Medical Subject Headings
MS	Multiple sclerosis
MWCNT	Multi-walled carbon nanotube
NOS	Newcastle–Ottawa Scale
NR	Not reported (in source publication)
PBM	Photobiomodulation
PBMT	Photobiomodulation therapy
PCC	Population–Concept–Context
PICOS	Population, Intervention, Comparator, Outcome, Study design
PPG	Photoplethysmography
PRISMA	Preferred Reporting Items for Systematic Reviews and Meta-Analyses
PRISMA-ScR	Preferred Reporting Items for Systematic Reviews and Meta-Analyses Extension for Scoping Reviews
QD	Quantum dot
RBC	Red blood cell
RCT	Randomized controlled trial
RNP	Redox-active nanoparticle
RNPN	pH-sensitive self-assembling reactive nanoparticle (subcutaneous)
RNPO	ROS-scavenging nanoparticle (oral, gastrointestinal-targeted)
RoB	Risk of bias
ROM	Range of motion
ROS	Reactive oxygen species
RSV	Resveratrol
SLN	Solid lipid nanoparticle
SOD	Superoxide dismutase
SpO_2_	Peripheral oxygen saturation
SR	Systematic review
SWCNT	Single-walled carbon nanotube
SYRCLE	Systematic Review Centre for Laboratory animal Experimentation
WADA	World Anti-Doping Agency
