# Supplementary material for: Applications of Nanotechnology in Athletic and Exercise Performance: A Scoping Review
Source: J Funct Morphol Kinesiol. 2026 Oct 13;11(4):416. doi: 10.3390/jfmk11040416 (PMC13692529; doi:10.3390/jfmk11040416)
Supplement: Supplementary file 1 [file jfmk-11-00416-s001.zip › jfmk-4559017-supplementary.pdf]

SUPPLEMENTARY MATERIAL S1

S1.1. Database 1: PubMed/MEDLINE

Search date: 12 June 2026

Interface: National Library of Medicine PubMed (https://pubmed.ncbi.nlm.nih.gov/)

Date range searched: Inception to 12 June 2026

Records retrieved: 298

Filters applied at database level: None (no date limits; no language restriction at search stage; language restriction applied post hoc at full-text eligibility screening)

Exact search string as executed:

("nanotechnology"[MeSH Terms] OR "nanoparticles"[MeSH Terms] OR "carbon nanotubes"[MeSH Terms] OR "quantum dots"[MeSH Terms] OR "nanosensors"[Title/Abstract] OR "nanomaterials"[Title/Abstract] OR "nanomaterial"[Title/Abstract] OR "photobiomodulation"[Title/Abstract] OR "Taopatch"[Title/Abstract] OR "nano-encapsulation"[Title/Abstract] OR "nanoencapsulation"[Title/Abstract] OR "nanocomposite"[Title/Abstract] OR "nanocomposites"[Title/Abstract] OR "graphene"[Title/Abstract] OR "quantum confinement"[Title/Abstract] OR "carbon nanotube"[Title/Abstract] OR "solid lipid nanoparticle"[Title/Abstract] OR "solid lipid nanoparticles"[Title/Abstract] OR "nanoparticle"[Title/Abstract] OR "quantum dot"[Title/Abstract] OR "nanosensor"[Title/Abstract]) AND ("athletic performance"[MeSH Terms] OR "exercise"[MeSH Terms] OR "athletes"[MeSH Terms] OR "physical fitness"[MeSH Terms] OR "sports"[MeSH Terms] OR "running"[MeSH Terms] OR "sports performance"[Title/Abstract] OR "elite athletes"[Title/Abstract] OR "physical performance"[Title/Abstract] OR "exercise performance"[Title/Abstract] OR "athletic performance"[Title/Abstract] OR "sport performance"[Title/Abstract] OR "exercise capacity"[Title/Abstract] OR "exercise tolerance"[Title/Abstract] OR "endurance performance"[Title/Abstract] OR "muscle performance"[Title/Abstract] OR "biomechanical performance"[Title/Abstract] OR "wearable sensor"[Title/Abstract] OR "wearable biosensor"[Title/Abstract] OR "sports equipment"[Title/Abstract] OR "exercise recovery"[Title/Abstract] OR "fatigue"[Title/Abstract])

MeSH terms verified against controlled vocabulary:

"Nanotechnology" — MeSH Unique ID: D036103

"Nanoparticles" — MeSH Unique ID: D053758

"Carbon Nanotubes" — MeSH Unique ID: D037742

"Quantum Dots" — MeSH Unique ID: D057166

"Athletic Performance" — MeSH Unique ID: D055380

"Exercise" — MeSH Unique ID: D015444

"Athletes" — MeSH Unique ID: D056352

"Physical Fitness" — MeSH Unique ID: D005082

"Sports" — MeSH Unique ID: D013177

"Running" — MeSH Unique ID: D012420

S1.2. Database 2: Scopus

Search date: 15 June 2026

Interface: Elsevier Scopus (https://www.scopus.com/)

Date range searched: Inception to 15 June 2026

Records retrieved: 287

Filters applied at database level: Document types: Article, Review, Conference paper, Book chapter; Language: All (language exclusion applied at full-text stage)

Exact search string as executed:

TITLE-ABS-KEY ("nanotechnology" OR "nanoparticle*" OR "carbon nanotube*" OR "quantum dot*" OR "nanosensor*" OR "nanomaterial*" OR "photobiomodulation" OR "Taopatch" OR "nano-encapsulat*" OR "nanoencapsulat*" OR "nanocomposit*" OR "graphene" OR "quantum confinement" OR "solid lipid nanoparticle*" OR "single-walled carbon nanotube*" OR "SWCNT" OR "multi-walled carbon nanotube*" OR "MWCNT" OR "graphene quantum dot*" OR "GQD" OR "photobiomodulation therapy" OR "PBMT" OR "low-level laser therapy" OR "LLLT" OR "wearable nanosensor*" OR "electrochemical biosensor*") AND TITLE-ABS-KEY ("athletic performance" OR "exercise" OR "athlete*" OR "sport* performance" OR "elite athlete*" OR "physical performance" OR "exercise performance" OR "exercise capacity" OR "exercise tolerance" OR "endurance performance" OR "endurance exercise" OR "muscle performance" OR "muscle fatigue" OR "running performance" OR "cycling performance" OR "sports equipment" OR "sports biomechanic*" OR "wearable sensor*" OR "sweat monitor*" OR "exercise recovery" OR "time to exhaustion" OR "VO2max" OR "oxidative stress" OR "antioxidant supplement*" OR "postural control" OR "balance" OR "proprioception" OR "biomechanical*" OR "sport*")

Field tags used: TITLE-ABS-KEY (searches Title, Abstract, and Author Keywords fields simultaneously)

Wildcard character: Asterisk (*) used for right-hand truncation to capture plural forms and variant endings

S1.3. Database 3: Web of Science, Core Collection

Search date: 15 June 2026

Interface: Clarivate Web of Science Core Collection (https://www.webofscience.com/)

Collections searched: Science Citation Index Expanded (SCIE); Social Sciences Citation Index (SSCI); Emerging Sources Citation Index (ESCI); Conference Proceedings Citation Index — Science (CPCI-S)

Date range searched: Inception to 15 June 2026

Records retrieved: 198

Filters applied at database level: None

Exact search string as executed:

TS=("nanotechnology" OR "nanoparticle*" OR "carbon nanotube*" OR "quantum dot*" OR "nanosensor*" OR "nanomaterial*" OR "photobiomodulation" OR "Taopatch" OR "nano-encapsulat*" OR "nanoencapsulat*" OR "nanocomposit*" OR "graphene" OR "quantum confinement" OR "solid lipid nanoparticle*" OR "single-walled carbon nanotube*" OR "SWCNT" OR "multi-walled carbon nanotube*" OR "MWCNT" OR "graphene quantum dot*" OR "GQD" OR "photobiomodulation therapy" OR "PBMT" OR "low-level laser therapy" OR "LLLT" OR "wearable nanosensor*" OR "electrochemical biosensor*") AND TS=("athletic performance" OR "exercise" OR "athlete*" OR "sport* performance" OR "elite athlete*" OR "physical performance" OR "exercise performance" OR "exercise capacity" OR "exercise tolerance" OR "endurance performance" OR "endurance exercise" OR "muscle performance" OR "muscle fatigue" OR "running performance" OR "cycling performance" OR "sports equipment" OR "sports biomechanic*" OR "wearable sensor*" OR "sweat monitor*" OR "exercise recovery" OR "time to exhaustion" OR "VO2max" OR "oxidative stress" OR "antioxidant supplement*" OR "postural control" OR "balance" OR "proprioception" OR "biomechanical*" OR "sport*")

Field tag: TS (Topic field — searches Title, Abstract, Author Keywords, and Keywords Plus)

Wildcard character: Asterisk (*) for right-hand truncation

S1.4. Manual Reference List Searching

Process: Reference lists of all 28 included studies and 12 pre-identified relevant narrative reviews of nanotechnology in sport (identified during preparatory reading) were manually screened by one reviewer (R.D.V.), with verification by a second reviewer (L.L.).

Records identified: 14 additional records

Search dates: 15–20 June 2026

S1.5. Databases Not Searched and Assessment of Likely Retrieval Impact

The following databases were not searched in this review. Future iterations must include these sources.

Database	Primary Domain	Likely Impact on Retrieval
SPORTDiscus (EBSCO)	Sports science, physical education, kinesiology	Moderate — additional athlete-specific performance studies, particularly in exercise physiology and sports training; many sports science journals are indexed here but not in PubMed/MEDLINE
Embase	Pharmacology, drug delivery, supplementation	Moderate to high — additional nano-supplementation, bioavailability, and pharmacokinetic studies relevant to nano-antioxidant domain; stronger indexing of European pharmaceutical literature
IEEE Xplore	Electrical engineering, wearable electronics, biosensors	High — substantial wearable sensor and nanosensor engineering literature indexed here would likely increase Stratum 4b and Stratum 2 wearable sensor evidence; IEEE Xplore is the primary indexing resource for this literature
Engineering Village / Compendex	Materials engineering, composites, mechanical engineering	Moderate to high — additional CNT-composite mechanical characterization studies; engineering conference proceedings not indexed elsewhere
CINAHL (EBSCO)	Nursing, allied health, rehabilitation	Low — limited relevance; some sports rehabilitation literature possibly missed
ClinicalTrials.gov and EU Clinical Trials Register	Trial registries (grey literature)	Unknown but potentially important — unpublished or ongoing RCTs on nanotechnological interventions in athletes could reduce publication bias; not searched in this review
ProQuest Dissertations and Theses	Grey literature	Low to moderate — graduate research in sports

		nanotechnology not yet published in peer-reviewed journals; not searched in this review

SUPPLEMENTARY MATERIAL S2

List of Contextual References

These references are cited in the Introduction (Section 1), Discussion (Section 4), or other explanatory sections of the manuscript to provide background context, mechanistic framing, regulatory information, or methodological benchmarks. They were NOT included among the 28 primary evidence map records (Strata 1–4) and were NOT required to satisfy the study eligibility criteria specified in Section 2.2. They are distinguished throughout the manuscript with the label [contextual reference]. This list is provided for complete transparency in accordance with Reviewer 2 Major Comment #8.

[CR-01] Feynman RP. There's plenty of room at the bottom. Engineering and Science. 1960;23(5):22–36.

Role in manuscript: Cited in Section 1 (Introduction) as the foundational historical reference establishing the conceptual basis of nanotechnology. Not a primary study; not subject to eligibility criteria.

[CR-02] Page MJ, McKenzie JE, Bossuyt PM, Boutron I, Hoffmann TC, Mulrow CD, et al. The PRISMA 2020 statement: an updated guideline for reporting systematic reviews. BMJ. 2021;372:n71. doi:10.1136/bmj.n71.

Role in manuscript: Cited in Section 2 (Methods) as a reporting standard reference. Not a primary study; cited for methodological compliance only.

[CR-03] Tricco AC, Lillie E, Zarin W, O'Brien KK, Colquhoun H, Levac D, et al. PRISMA Extension for Scoping Reviews (PRISMA-ScR): checklist and explanation. Ann Intern Med. 2018;169(7):467–473. doi:10.7326/M18-0850.

Role in manuscript: Cited in Section 2 (Methods) as the primary reporting standard for this systematic scoping review. Not a primary study.

[CR-04] Sterne JAC, Savović J, Page MJ, Elbers RG, Blencowe NS, Boutron I, et al. RoB 2: a revised tool for assessing risk of bias in randomized trials. BMJ. 2019;366:l4898. doi:10.1136/bmj.l4898.

Role in manuscript: Cited in Section 2.6 (Quality Assessment) as the source and validation reference for the RoB 2 tool. Not a primary study.

[CR-05] Wells G, Shea B, O'Connell D, Peterson J, Welch V, Losos M, Tugwell P. The Newcastle–Ottawa Scale (NOS) for Assessing the Quality of Non-Randomized Studies in Meta-Analysis. Ottawa Hospital Research Institute; 2000. Available from: http://www.ohri.ca/programs/clinical_epidemiology/oxford.asp.

Role in manuscript: Cited in Section 2.6 (Quality Assessment) as the source and validation reference for the NOS tool. Not a primary study.

[CR-06] Hooijmans CR, Rovers MM, de Vries RB, Leenaars M, Ritskes-Hoitinga M, Langendam MW. SYRCLE's risk of bias tool for animal studies. BMC Med Res Methodol. 2014;14:43. doi:10.1186/1471-2288-14-43.

Role in manuscript: Cited in Section 2.6 (Quality Assessment) as the source and validation reference for the SYRCLE tool. Not a primary study.

[CR-07] Shea BJ, Reeves BC, Wells G, Thuku M, Hamel C, Moran J, et al. AMSTAR 2: a critical appraisal tool for systematic reviews that include randomized or non-randomized studies of healthcare interventions, or both. BMJ. 2017;358:j4008. doi:10.1136/bmj.j4008.

Role in manuscript: Cited in Section 2.6 (Quality Assessment) as the source and validation reference for the AMSTAR-2 tool. Not a primary study.

[CR-08] Li S, Fasibe B, Laher I. Potential harms of supplementation with high doses of antioxidants in athletes. J Exerc Sci Fit. 2022;20(4):269–275. doi:10.1016/j.jesf.2022.06.001.

Role in manuscript: Cited in Sections 3.3.2 and 4.1.2 as contextual evidence for the paradoxical impairment of training adaptation by high-dose antioxidants; provides mechanistic framing for nano-antioxidant discussion. Not included among the 28 primary evidence map records as it does not directly evaluate a nanotechnological intervention.

[CR-09] Abdellatif AAH, Younis MA, Alsharidah M, Al Rugaie O, Tawfeek HM. Biomedical applications of quantum dots: overview, challenges, and clinical potential. Int J Nanomedicine. 2022;17:1951–1970. doi:10.2147/IJN.S357532.

Role in manuscript: Cited in Section 3.4 (Cross-Domain Translational Considerations) as contextual evidence on QD cytotoxicity and clinical translation barriers. Cross-domain contextual reference; not included in Table 2 as a primary evidence map record.

[CR-10] Nomura H, Takanashi S, Tanaka M, Haniu H, Aoki K, Okamoto M, et al. Specific biological responses of the synovial membrane to carbon nanotubes. Sci Rep. 2015;5:14314. doi:10.1038/srep14314.

Role in manuscript: Cited in Section 3.4 (Cross-Domain Translational Considerations) as contextual safety evidence for CNT biocompatibility in musculoskeletal tissues. Cross-domain contextual reference; not included in Table 2 as a primary evidence map record.

[CR-11] Li H, Zhai X, Yang Z, Tang X, Wang J, Qiu X. Modification and application of sports rehabilitation materials based on conjugated materials. Front Chem. 2023;11:1294152. doi:10.3389/fchem.2023.1294152.

Role in manuscript: Cited in Section 3.4 (Cross-Domain Translational Considerations) as contextual evidence on nano-composite rehabilitation materials. Cross-domain contextual reference; not included in Table 2 as a primary evidence map record.

[CR-12] Fernandes MSS, Fidelis DEDS, Aidar FJ, Badicu G, Greco G, Cataldi S, et al. Coenzyme Q10 supplementation in athletes: a systematic review. Nutrients. 2023;15(18):3990. doi:10.3390/nu15183990.

Role in manuscript: Cited in Sections 3.4 and 4.1.2 as a contextual evidence benchmark for conventional CoQ10 supplementation in athletes, providing the translational rationale for nano-encapsulated CoQ10. Used as an evidence benchmark, not as a primary nanotechnology study; not included in Table 2.

[CR-13] Mansuriya BD, Altintas Z. Graphene quantum dot-based electrochemical immunosensors for biomedical applications. Materials (Basel). 2019;13(1):96. doi:10.3390/ma13010096.

Role in manuscript: Cited in Section 3.4 (Cross-Domain Translational Considerations) as contextual evidence for GQD biosensor detection limits. Cross-domain contextual reference; not included in Table 2.

[CR-14] De Vitis R, Lombardi L, Guzzini M, Militerno A, Taccardo G, Passiatore M. Why hand-wrist bandaging could improve performance in elite soccer players? A scoping review on the biomechanical rationale of upper limb role in kicking. Sports (Basel). 2026;14(5):189. doi:10.3390/sports14050189.

Role in manuscript: Cited in Sections 4.1.1 and 4.2 as a contextual reference illustrating the principle of marginal gains through peripheral biomechanical interventions; cited to support analogical reasoning, not as primary evidence for any nanotechnological outcome.

[CR-15] Smoliga JM, Blanchard O. Enhancing the delivery of resveratrol in humans: if low bioavailability is the problem, what is the solution? Molecules. 2014;19(11):17154–17172. doi:10.3390/molecules191117154.

Role in manuscript: Cited in Section 4.1.2 and 4.2 as contextual pharmacokinetic evidence for the bioavailability limitations of conventional oral resveratrol, supporting the translational rationale for nanoparticle encapsulation.

[CR-16] Talebi S, Pourgharib Shahi MH, Zeraattalab-Motlagh S, Mohammadi H, Moradi S, Naeini F, et al. The effects of coenzyme Q10 supplementation on biomarkers of exercise-induced muscle damage, physical performance, and oxidative stress: a GRADE-assessed systematic review and dose-response meta-analysis of randomized controlled trials. Clin Nutr ESPEN. 2024;60:122–134. doi:10.1016/j.clnesp.2023.12.030.

Role in manuscript: Cited in Section 4.1.2 as contextual dose-response evidence for CoQ10 supplementation, supporting the translational rationale for nano-encapsulation to enhance dosimetric precision. Contextual benchmark; not a nanotechnology study.

[CR-17] Chen J, Ding J, Han C, Chen M, Zhao Z, Chen X, et al. Effects of different antioxidants on exercise-induced oxidative stress and muscle damage in athletes: a systematic review and meta-analysis. BMC Sports Sci Med Rehabil. 2025;17(1):328. doi:10.1186/s13102-025-01328-x.

Role in manuscript: Cited in Section 4.1.2 as contextual evidence characterizing the partial and heterogeneous efficacy of conventional antioxidant supplementation in athletes; supports the translational rationale for nano-antioxidant strategies. Not a nanotechnology study.

[CR-18] Assalve G, Lunetti P, Di Cagno A, Marsigliante S, Marsigliante G, Sanapo F, et al. Advanced wearable devices for monitoring sweat biochemical markers in athletic performance: a comprehensive review. Biosensors (Basel). 2024;14(12):574. doi:10.3390/bios14120574.

Role in manuscript: Cited in Section 4.1.3 as contextual evidence identifying primary target analytes for wearable athlete monitoring; supports interpretation of wearable nanosensor domain findings.

[CR-19] Yang G, Hong J, Park SB. Wearable device for continuous sweat lactate monitoring in sports: a narrative review. Front Physiol. 2024;15:1376801. doi:10.3389/fphys.2024.1376801.

Role in manuscript: Cited in Section 4.1.3 as contextual evidence on sweat lactate monitoring challenges; provides the translational context for interpreting wearable nanosensor applications.

[CR-20] Niazi SK. Nanomaterial-enabled physical rehabilitation: mechanisms, evidence, and translational pathways. Front Rehabil Sci. 2026;7:1763655. doi:10.3389/fresc.2026.1763655.

Role in manuscript: Cited in Section 4.1.3 as contextual evidence for nanomaterial-mediated enhancement of musculoskeletal repair; reinforces biological plausibility while acknowledging translational limitations.

[CR-21] Leal-Junior ECP, Lopes-Martins RÁB, Bjordal JM. Clinical and scientific recommendations for the use of photobiomodulation therapy in exercise performance enhancement and post-exercise recovery:

current evidence and future directions. Braz J Phys Ther. 2019 Jan-Feb;23(1):71-75. doi: 10.1016/j.bjpt.2018.12.002.

Role in manuscript: Cited in Section 4.1.4 as contextual evidence providing foundational PBM exercise performance data against which nanotechnology-based photoemission systems should be benchmarked. This is a distinct publication from the Leal-Junior 2013 systematic review included in Table 2 (Study 13) and contributes contextual rather than primary evidence map data.

[CR-22] Wang Q, Sun J, Su Z, Xiang L. Combining exercise and nanoparticle-based therapies: a review of physiological synergies and pharmacological potential. Front Pharmacol. 2026;16:1679552. doi:10.3389/fphar.2026.1679552.

Role in manuscript: Cited in Section 4.3 as contextual mechanistic evidence on the potential physiological environment created by exercise for nanoparticle uptake. The authors explicitly characterize the described mechanisms as hypothetical and not clinically established in athlete populations; cited accordingly.

SUPPLEMENTARY MATERIAL S3

Preferred Reporting Items for Systematic reviews and Meta-Analyses extension for Scoping Reviews (PRISMA-ScR) Checklist

SECTION	ITEM	PRISMA-ScR CHECKLIST ITEM	REPORTED ON PAGE #
**TITLE**			
Title	1	Identify the report as a scoping review.	Title page: "A Systematic Scoping Review"
**ABSTRACT**			
Structured summary	2	Provide a structured summary that includes (as applicable): background, objectives, eligibility criteria, sources of evidence, charting methods, results, and conclusions that relate to the review questions and objectives.	Abstract page 2
**INTRODUCTION**			
Rationale	3	Describe the rationale for the review in the context of what is already known. Explain why the review questions/objectives lend themselves to a scoping review approach.	Section 1 (Introduction), paragraph 5; Section 2.1
Objectives	4	Provide an explicit statement of the questions and objectives being addressed with reference to their key elements (e.g., population or participants, concepts, and context) or other relevant key elements used to conceptualize the review questions and/or objectives.	Section 1 (final paragraph); Section 2.2 (PCC Framework)
**METHODS**			
Protocol and registration	5	Indicate whether a review protocol exists; state if and where it can be accessed (e.g., a Web address); and if	Section 2.1: explicitly states that no prospective protocol registration on PROSPERO

SECTION	ITEM	PRISMA-ScR CHECKLIST ITEM	REPORTED ON PAGE #
		available, provide registration information, including the registration number.	or OSF was completed; limitation acknowledged and discussed in Section 4.5
Eligibility criteria	6	Specify characteristics of the sources of evidence used as eligibility criteria (e.g., years considered, language, and publication status), and provide a rationale.	Section 2.2 (full PCC framework; levelled eligibility criteria; Table 1); inclusion and exclusion criteria listed explicitly
Information sources*	7	Describe all information sources in the search (e.g., databases with dates of coverage and contact with authors to identify additional sources), as well as the date the most recent search was executed.	Section 2.3; Supplementary Material S1; grey literature and trial registry non-searching acknowledged in Section 2.3 and Section 4.5
Search	8	Present the full electronic search strategy for at least 1 database, including any limits used, such that it could be repeated.	Section 2.3 (PubMed/MEDLINE string); Supplementary Material S1 (all three databases, exact strings)
Selection of sources of evidence†	9	State the process for selecting sources of evidence (i.e., screening and eligibility) included in the scoping review.	Section 2.4; PRISMA-ScR flow diagram (Figure 1)
Data charting process‡	10	Describe the methods of charting data from the included sources of evidence (e.g., calibrated forms or forms that have been tested by the team before their use, and whether data charting was done independently or in duplicate) and any processes for obtaining and confirming data from investigators.	Section 2.5; standardized data extraction form described; form available from corresponding author
Data items	11	List and define all variables for which data were sought and any assumptions and simplifications made.	Section 2.5; Table 2 column headings
Critical appraisal of individual sources of evidence§	12	If done, provide a rationale for conducting a critical appraisal of included sources of evidence; describe the methods used and how this information was used in any data synthesis (if appropriate).	Section 2.6; quality appraisal tools specified per stratum; limitations of narrative frameworks acknowledged; Section 4.5
Synthesis of results	13	Describe the methods of handling and summarizing the data that were charted.	Section 2.7; narrative synthesis rationale; domain-specific presentation approach; Table 3 evidence summary matrix
**RESULTS**			
Selection of sources of evidence	14	Give numbers of sources of evidence screened, assessed for eligibility, and included in the review, with reasons for exclusions at each stage, ideally using a flow diagram.	Section 3.1; Figure 1 (PRISMA-ScR flow diagram)
Characteristics of sources of evidence	15	For each source of evidence, present characteristics for which data were charted and provide the citations.	Section 3.2; Table 2 (abbreviated, main text);

SECTION	ITEM	PRISMA-ScR CHECKLIST ITEM	REPORTED ON PAGE #
			Supplementary Material S4 (full Table 2)
Critical appraisal within sources of evidence	16	If done, present data on critical appraisal of included sources of evidence (see item 12).	Table 2 (QA Tool and QA Result columns); Section 3.3 domain-specific narratives; Table 3 summary matrix
Results of individual sources of evidence	17	For each included source of evidence, present the relevant data that were charted that relate to the review questions and objectives.	Section 3.3 (domain-specific findings); Table 2; Supplementary Material S4
Synthesis of results	18	Summarize and/or present the charting results as they relate to the review questions and objectives.	Section 3.3; Section 4.1; Table 3
**DISCUSSION**			
Summary of evidence	19	Summarize the main results (including an overview of concepts, themes, and types of evidence available), link to the review questions and objectives, and consider the relevance to key groups.	Section 4.1 (sub-sections 4.1.1–4.1.5); Section 4.2; Section 4.3
Limitations	20	Discuss the limitations of the scoping review process.	Section 4.5 (nine explicitly enumerated limitations)
Conclusions	21	Provide a general interpretation of the results with respect to the review questions and objectives, as well as potential implications and/or next steps.	Section 4.6; Section 5
**FUNDING**			
Funding	22	Describe sources of funding for the included sources of evidence, as well as sources of funding for the scoping review. Describe the role of the funders of the scoping review.	Funding statement: "This research received no external funding"; conflicts of interest declared as none

JBI = Joanna Briggs Institute; PRISMA-ScR = Preferred Reporting Items for Systematic reviews and Meta-Analyses extension for Scoping Reviews.

* Where *sources of evidence* (see second footnote) are compiled from, such as bibliographic databases, social media platforms, and Web sites.

† A more inclusive/heterogeneous term used to account for the different types of evidence or data sources (e.g., quantitative and/or qualitative research, expert opinion, and policy documents) that may be eligible in a scoping review as opposed to only studies. This is not to be confused with *information sources* (see first footnote).

‡ The frameworks by Arksey and O'Malley (6) and Levac and colleagues (7) and the JBI guidance (4, 5) refer to the process of data extraction in a scoping review as data charting.

§ The process of systematically examining research evidence to assess its validity, results, and relevance before using it to inform a decision. This term is used for items 12 and 19 instead of "risk of bias" (which is more applicable to systematic reviews of interventions) to include and acknowledge the various sources of evidence that may be used in a scoping review (e.g., quantitative and/or qualitative research, expert opinion, and policy document).

From: Tricco AC, Lillie E, Zarin W, O'Brien KK, Colquhoun H, Levac D, et al. PRISMA Extension for Scoping Reviews (PRISMAScR): Checklist and Explanation. Ann Intern Med. 2018;169:467–473. .

SUPPLEMENTARY MATERIAL S4

Full Data Extraction Table (Complete Table 2)

This full table reproduces the complete data extraction for all 28 included records, with the following additions compared to the abbreviated Table 2 in the main manuscript: (a) standardized participant characteristics columns (training status, sex, age, sport where reported); (b) effect estimates with 95% CI or SD where available in source publication; (c) corrected reference numbers where discrepancies between table citation numbers and reference list numbers were identified in the original submission. Corrections are noted in the footnotes. Evidence strata labels (Stratum 1–4) replace "Level 1–4" as applied throughout the revised manuscript.

STRATUM 1 — DIRECT CONTROLLED EVIDENCE (n = 2)

Study 1

First Author, Year, Corrected Reference: Ko, 2023 [Ref. 21]

Country: Republic of Korea

Domain: CNT-based sports equipment

Study Design: Controlled within-subject crossover trial (acute, single-session)

Evidence Stratum: Stratum 1 (lower boundary — see notes)

Training Status: Healthy adult males, not formally classified as elite or trained athletes

Sex: Male (100%)

Age: Not reported in source publication (NR)

Sport: Not sport-specific; healthy university-affiliated males

Sample Size: n = 30

Nanotechnological Intervention: Carbon fiber insole (CFI) incorporating CNT-reinforced composite

Comparator: Conventional standard insole (same footwear, no CNT component)

Primary Outcome Measures: Vertical jump height (cm); agility performance (s); sprint speed (s)

Key Quantitative Results: Vertical jump: +1.5% (magnitude NR in source as absolute value with 95% CI; $p < 0.05$); Agility: +1.0% improvement ($p < 0.05$); Sprint speed: no statistically significant difference ($p > 0.05$)

Effect estimate availability: Percentage change and p-values reported; 95% CI not provided in source publication

Sample Size Calculation Reported: No

MCID/SWC Threshold Reported: No

Quality Appraisal Tool: Cochrane Risk of Bias 2 (RoB 2)

QA Domain Judgements: D1 (Randomization): Low concern; D2 (Deviations from intervention): Low concern; D3 (Missing outcome data): Low concern; D4 (Outcome measurement): Some concerns — outcome assessor blinding insufficiently described; D5 (Reported result selection): Some concerns — no pre-registered protocol; selective reporting not excludable

Overall RoB 2 Classification: Some concerns

Authors' Stated Limitations: Acute crossover design limits generalizability; no elite athlete population; no long-term data

Key Caveats for Interpretation: Population not elite athletes (Stratum 1 lower boundary; proof-of-concept only); no MCID or SWC reported; percentage change alone insufficient for practical significance judgment; macro-composite rather than purely nanoscale intervention

Study 2

First Author, Year, Corrected Reference: Malchiodi Albedi, 2018 [Ref. 11]

Country: Italy

Domain: PBM nanotechnology (Taopatch®)

Study Design: Parallel-group randomized controlled trial

Evidence Stratum: Stratum 1

Training Status: Healthy subjects; not athletes

Sex: NR (not reported in source publication)

Age: NR

Sport: N/A (non-athletic population)

Sample Size: n = 40

Nanotechnological Intervention: Taopatch® CE Class I photoselective nanomaterial patch; body-heat-activated; manufacturer-claimed emission 400–1400 nm

Comparator: Sham device (identical appearance; no active nanomaterial component)

Primary Outcome Measures: Postural control parameters (stabilometry); centre-of-pressure displacement measures

Key Quantitative Results: Significant improvement in postural control parameters in Taopatch® vs. sham group ($p < 0.05$); specific effect size and 95% CI not provided in source

Effect estimate availability: p-value only; no 95% CI or effect size reported in source

Quality Appraisal Tool: Cochrane Risk of Bias 2 (RoB 2)

QA Domain Judgements: D1 (Randomization): Some concerns — randomisation process not fully described; D2 (Deviations from intervention): Some concerns — feasibility of sham blinding not documented; D3 (Missing outcome data): Low concern; D4 (Outcome measurement): High concern — outcome assessor blinding insufficient; D5 (Reported result selection): High concern — no pre-registered protocol; selective reporting not excludable

Overall RoB 2 Classification: High risk of bias

Authors' Stated Limitations: Small sample; non-athlete population; authors qualified results as preliminary data; nanomaterial composition and emission dose not independently characterised

Key Caveats for Interpretation: Non-athlete population; nanomaterial composition not independently characterised; emission dose not quantified; sham blinding feasibility not documented; high RoB; not directly applicable to elite athletic performance without further specific evidence

STRATUM 2 — OBSERVATIONAL/QUASI-EXPERIMENTAL HUMAN EVIDENCE (n = 7)

Study 3

First Author, Year, Corrected Reference: Messina, 2022 [Ref. 12]

Country: Italy

Domain: PBM nanotechnology (Taopatch®)

Study Design: Prospective observational cohort study (single arm, no separate control group)

Evidence Stratum: Stratum 2

Training Status: Clinical population (multiple sclerosis patients); not athletes

Sex: 8 male, 12 female

Age: NR (range not specified in source)

Sport: N/A

Sample Size: n = 20

Nanotechnological Intervention: Taopatch® combined with a structured proprioceptive rehabilitation protocol

Comparator: Pre-intervention baseline only (no separate control group; single-arm pre-post design)

Primary Outcome Measures: Baropodometric balance parameters; bilateral handgrip strength (kg); serum 25-hydroxyvitamin D (nmol/L)

Key Quantitative Results: Significant improvement in baropodometric balance ($p < 0.05$); Handgrip strength right: $p = 0.023$; Handgrip strength left: $p = 0.021$; Vitamin D trend to increase (NS; p value NR); specific mean differences and 95% CI NR in source

Effect estimate availability: p-values reported; mean differences and 95% CI not reported in source

NOS Score: 4★/9 (high risk of bias)

NOS Domain Assessment: Selection: 2★; Comparability: 0★ (no control group); Outcome: 2★

Authors' Stated Limitations: No control group; small sample; combined intervention (Taopatch® + rehabilitation); effects not separable

Key Caveats for Interpretation: No control group — effects of Taopatch® not separable from rehabilitation protocol; non-athlete clinical population; mechanism by which topically applied PBM device would produce measurable increase in circulating 25-OH vitamin D unexplained; requires independent replication

Study 4

First Author, Year, Corrected Reference: Carbonari, 2021 [Ref. 24]

Country: Italy

Domain: PBM nanotechnology (Taopatch®)

Study Design: Retrospective cross-over study

Evidence Stratum: Stratum 2

Training Status: Competitive athletes

Sex: NR

Age: NR

Sport: Mixed competitive sports (specific disciplines NR)

Sample Size: n = 25

Nanotechnological Intervention: Taopatch® device applied at defined anatomical points

Comparator: Occlusal splint (unrelated mechanical dental intervention used as active comparator condition; no true sham control)

Primary Outcome Measures: Muscular performance indices; postural balance; posture parameters (specific tools NR in source)

Key Quantitative Results: Both interventions produced measurable improvements in measured parameters; no differential effect between Taopatch® and occlusal splint specifically reported with 95% CI; p-values reported as significant for within-group changes

Effect estimate availability: Within-group p-values; no between-group effect size or 95% CI reported

NOS Score: 4★/9 (high risk of bias)

NOS Domain Assessment: Selection: 2★; Comparability: 1★; Outcome: 1★

Authors' Stated Limitations: Retrospective design; unblinded; absence of true sham control

Key Caveats for Interpretation: Absence of true sham/placebo control — placebo effect not excludable; retrospective design; both Taopatch® and an unrelated dental mechanical intervention (occlusal splint) produced similar improvements, raising strong possibility of placebo response; results require replication in blinded RCT

Study 5

First Author, Year, Corrected Reference: Amato, 2021 [Ref. 26]

Country: Italy

Domain: PBM nanotechnology (Taopatch®)

Study Design: Pilot pre-post study (non-RCT, single arm)

Evidence Stratum: Stratum 2

Training Status: Clinical population (multiple sclerosis patients); not athletes; study conducted during COVID-19 pandemic lockdown

Sex: NR

Age: NR

Sport: N/A

Sample Size: n = 10

Nanotechnological Intervention: Taopatch® combined with home-based exercise training protocol

Comparator: Pre-intervention baseline only

Primary Outcome Measures: Sedentary behaviour (daily activity); biochemical markers (specific markers NR in source abstract)

Key Quantitative Results: Decrease in sedentary time reported; changes in biochemical markers reported; specific effect sizes and 95% CI NR

Effect estimate availability: Directional results reported; quantitative effect sizes NR in source

NOS Score: 3★/9 (likely high risk of bias)

NOS Domain Assessment: Selection: 2★; Comparability: 0★; Outcome: 1★

Authors' Stated Limitations: No control group; very small sample; combined intervention

Key Caveats for Interpretation: Effects of Taopatch® not separable from exercise training component; non-athlete clinical population; very small sample (n = 10); retained for completeness of Taopatch® evidence synthesis and mechanistic framing only; not applicable to elite athletic populations

Study 6

First Author, Year, Corrected Reference: Wyckoff, 2025 [Ref. 23]

Country: USA

Domain: Wearable nanosensors

Study Design: Prospective sensor validation study (human validation cohort, between-group material comparison)

Evidence Stratum: Stratum 2

Training Status: Healthy subjects (non-athlete; not sport-specific); study population for sensor validation

Sex: NR

Age: NR

Sport: N/A (biomechanical validation context applicable to sports rehabilitation)

Sample Size: n = 20 (n = 10 per material group: MWCNT vs. graphene nanosheet)

Nanotechnological Intervention: Multi-walled carbon nanotube (MWCNT) elastic fabric motion tape sensors

Comparator: Graphene nanosheet tape sensors (alternative nanomaterial configuration)

Primary Outcome Measures: Low back movement characterization accuracy (forward flexion, lateral bending, axial rotation); signal stability under high-strain conditions; sensor performance metrics

Key Quantitative Results: MWCNT sensors accurately characterized all three low back movement types; MWCNT > graphene nanosheet for signal stability under high-strain conditions; specific accuracy percentages and 95% CI available in source publication — see source for full metrics

Effect estimate availability: Quantitative sensor performance metrics reported; 95% CI available in source

NOS Score: 8★/9 (high quality)

NOS Domain Assessment: Selection: 3★; Comparability: 2★; Outcome: 3★

Authors' Stated Limitations: Validation study design; not an athlete performance trial; translation to competitive performance contexts requires further validation

Key Caveats for Interpretation: Technical feasibility and physiological/biomechanical validation stage; direct athletic performance benefit not demonstrated; healthy non-athlete population

Study 7

First Author, Year, Corrected Reference: Campoli, 2026 [Ref. 25]

Country: Italy

Domain: PBM nanotechnology (Taopatch®)

Study Design: Pilot quasi-experimental study (non-randomized in nanotechnology allocation dimension)

Evidence Stratum: Stratum 2

Training Status: Elderly hearing aid users; non-athletic, elderly population

Sex: 19 male, 21 female

Age: Mean 77.3 ± 15.6 years

Sport: N/A

Sample Size: n = 40

Nanotechnological Intervention: Taopatch® photoselective nanomaterial patch integrated into hearing aid device (multimodal intervention)

Comparator: Hearing aid alone (standard condition)

Primary Outcome Measures: Posturographic balance parameters

Key Quantitative Results: Preliminary improvements in balance parameters reported; specific effect sizes and 95% CI NR in source

Effect estimate availability: Directional findings; quantitative effect sizes NR

NOS Score: 3★/9 (likely high risk of bias)

NOS Domain Assessment: Selection: 2★; Comparability: 0★; Outcome: 1★

Authors' Stated Limitations: Non-randomized; multimodal intervention; small non-athletic elderly population

Key Caveats for Interpretation: Multimodal intervention prevents attribution of any observed effect to the Taopatch® component; non-athletic, elderly population (mean age 77.3 y); no translational relevance to elite athlete performance without independent confirmation of nanotechnological effect contribution

Study 8

First Author, Year, Corrected Reference: Akiyama, 2022 [Ref. 33]

Country: Japan

Domain: Externally applied nanomaterial (IFMC)

Study Design: Acute treadmill exercise study (human, within-subject repeated measures)

Evidence Stratum: Stratum 2

Training Status: Athletic club students (university athletic club members; trained, not elite)

Sex: NR

Age: NR (university students implied)

Sport: Mixed university athletic club sports

Sample Size: n = 18 (106 total measurements across repeated testing sessions)

Nanotechnological Intervention: Externally applied, natural-mineral-based novel nanomaterial IFMC (Japanese hot-spring-derived; components: haematite, olivine, rhodolite, zincite; applied externally to skin surface)

Comparator: Sham/control condition

Primary Outcome Measures: Heart rate (HR) during aerobic exercise; VO_2; VO_2max; CO_2 production; respiratory quotient (RQ)

Key Quantitative Results: Significant reduction in HR elevation in IFMC group ($p < 0.05$); no statistically significant difference in VO_2, VO_2max, CO_2 production, or RQ; HR difference expressed as mean reduction NR in source with 95% CI

Effect estimate availability: p-values reported; mean differences and 95% CI NR

NOS Score: 4★/9 (moderate risk of bias)

NOS Domain Assessment: Selection: 2★; Comparability: 1★; Outcome: 1★

Authors' Stated Limitations: Small sample; acute single-session design; precise mechanism under investigation

Key Caveats for Interpretation: Preliminary evidence of cardiovascular function effect; primary performance outcomes (VO_2max) not significantly altered; external application mechanism poorly understood; requires replication in larger controlled study

Study 9

First Author, Year, Corrected Reference: Li S, 2023 [Ref. 29]

Country: China/USA (multi-centre engineering and human validation study)

Domain: QD-based wearable biosensing

Study Design: Engineering development + human validation study

Evidence Stratum: Stratum 2

Training Status: Healthy subjects (sensor validation participants; not athletes)

Sex: NR

Age: NR

Sport: N/A

Sample Size: n = 5 (human validation component)

Nanotechnological Intervention: Cu-In-Se quantum dot-based wearable photoplethysmography (PPG) photodetector (heavy-metal-free formulation)

Comparator: Conventional reference monitoring device (PPG-based)

Primary Outcome Measures: Heart rate (HR) fidelity; SpO_2 monitoring accuracy; mechanical flexibility of device; photon detection performance metrics

Key Quantitative Results: High-fidelity PPG signal performance; mechanical flexibility adequate for wearable application; HR monitoring accuracy comparable to reference; heavy-metal-free formulation addresses CdSe/CdTe cytotoxicity barrier; full quantitative metrics available in source

Effect estimate availability: Engineering performance metrics reported in full; clinical effect estimates not applicable (technical validation study)

NOS Score: 5★/9 (moderate quality)

NOS Domain Assessment: Selection: 3★; Comparability: 1★; Outcome: 1★

Authors' Stated Limitations: Very small human validation sample (n = 5); not validated in athletic exercise contexts; engineering validation only

Key Caveats for Interpretation: Technical feasibility stage; n = 5 human validation; not validated in exercise or athletic monitoring context; no direct athletic performance outcome measured

STRATUM 3 — TRANSLATIONAL ANIMAL EXERCISE MODELS (Nano-antioxidant domain only; n = 3)

Stratum 3 was restricted a priori to the nano-supplementation domain. The restriction decision was established during protocol development prior to search commencement (see Section 2.2 and Supplementary Material S2 rationale). All three studies used rodent exercise models; results must not be directly extrapolated to elite human athletes without controlled human trial confirmation.

Study 10

First Author, Year, Reference: Toriumi, 2021 [Ref. 6]

Country: Japan

Domain: Nano-antioxidant supplementation

Study Design: In vivo controlled animal exercise experiment

Evidence Stratum: Stratum 3

Animal Model: Male Wistar rats; validated treadmill exhaustion model

Sample Size per Group: n ≈ 7

Nanotechnological Intervention: RNPN — pH-sensitive self-assembling reactive nanoparticle antioxidant (subcutaneous administration)

Comparator: Vehicle control (subcutaneous)

Primary Outcome Measures: Treadmill running time to exhaustion (min; 40 m/min); post-exercise circulating red blood cell (RBC) count

Key Quantitative Results: Running time ≈ +50% increase (60 min → 90 min; $p < 0.05$); significant attenuation of post-exercise RBC reduction ($p < 0.05$); 95% CI NR in source

SYRCLE Assessment: Unclear risk — sequence generation, allocation concealment, and outcome assessor blinding inadequately reported

Authors' Stated Limitations: Rodent model; subcutaneous administration route has no clinically realistic athlete equivalent; small group sizes

Key Caveats for Interpretation: Dual translational barrier: rodent-to-human species extrapolation AND subcutaneous administration route with no clinically realistic athlete equivalent; preliminary authors' priority claim (characterised as "first nanoparticle-type antioxidant study in exercise performance") is the authors' own assertion, not independently corroborated

Study 11

First Author, Year, Reference: Toriumi, 2023 [Ref. 7]

Country: Japan

Domain: Nano-antioxidant supplementation

Study Design: In vivo controlled animal exercise experiment (dose-escalation design)

Evidence Stratum: Stratum 3

Animal Model: Male C57BL/6J mice; treadmill exhaustion model

Sample Size per Group: n = 7–8

Nanotechnological Intervention: RNPO — oral ROS-scavenging nanoparticle selectively targeting the gastrointestinal (GI) tract (dose-escalation: multiple dose groups)

Comparator: Free antioxidant TEMPOL (oral, equivalent dose); vehicle control

Primary Outcome Measures: Running time to exhaustion; GI mucosal integrity; plasma lipopolysaccharide (LPS) concentration

Key Quantitative Results: Running time extended in dose-dependent manner ($p < 0.05$); TEMPOL reduced running time at high dose; GI mucosa protected in RNPO group; plasma LPS significantly lower in RNPO vs. vehicle ($p < 0.05$); specific mean differences and 95% CI NR in source

SYRCLE Assessment: Unclear risk — multiple domains insufficiently reported (allocation concealment, blinding, outcome reporting)

Authors' Stated Limitations: Animal model; dose-escalation design in mice; precise human dose equivalents unclear

Key Caveats for Interpretation: Oral route is translationally relevant; demonstrated mechanistic pathway (GI mucosa protection against exercise-induced GI syndrome); nano-formulation superior to equivalent-dose conventional antioxidant; however, full human translation (dose, frequency, safety) unstudied

Study 12

First Author, Year, Reference: Qin, 2020 [Ref. 8]

Country: China

Domain: Nano-antioxidant supplementation

Study Design: In vivo controlled animal exercise experiment

Evidence Stratum: Stratum 3

Animal Model: Male ICR mice; exhaustive running model

Sample Size per Group: n = 10

Nanotechnological Intervention: RSV-SLN — resveratrol-loaded solid lipid nanoparticles (oral administration)

Comparator: Free resveratrol (oral, equivalent dose); vehicle control

Primary Outcome Measures: Exhaustive running time (min); SOD activity (U/mL); MDA concentration (nmol/mL); SIRT1/autophagy pathway markers (LC3-II, p62)

Key Quantitative Results: Running time significantly prolonged vs. free RSV ($p < 0.05$); SOD significantly increased ($p < 0.05$); MDA significantly decreased ($p < 0.05$); SIRT1/autophagy pathway identified as mechanistic mediator; specific mean differences and 95% CI NR in source

SYRCLE Assessment: Unclear risk — multiple domains insufficiently reported

Authors' Stated Limitations: Animal model; single-dose comparison; human pharmacokinetic and safety data absent

Key Caveats for Interpretation: Nano-encapsulation overcomes poor oral bioavailability of free resveratrol; SIRT1/autophagy mechanistic pathway provides novel biological framework; direct translation to humans requires PK/PD studies and safety data prior to athlete trials

STRATUM 4a — TRUE SYSTEMATIC REVIEWS AND META-ANALYSES (n = 5)

Study 13

First Author, Year, Reference: Leal-Junior, 2015 [Ref. 32/49 — see footnote A]

Country: Brazil/International

Domain: PBM — contextual framework for exercise performance

Study Design: Systematic review + meta-analysis (PEDro ≥ 6/10 quality threshold for inclusion)

Evidence Stratum: Stratum 4a

Included Studies: 16 RCTs retrieved; 13 included in meta-analysis (PEDro ≥ 6/10); total participant n NR per included study in source abstract

Intervention Category: Phototherapy (LLLT + LED); pre-, during, and post-exercise application

Primary Outcome Measures: Number of repetitions to failure; time to exhaustion (s); creatine kinase (CK) activity

Key Quantitative Results: ↑ Time to exhaustion: MD +4.12 s (95% CI 1.21–7.02; $p < 0.005$); ↑ Repetitions to failure: MD +5.47 (95% CI 2.35–8.59; $p < 0.0006$); most consistent results: red/infrared wavelengths; pre-exercise application; 50–200 mW; 5–6 J/point; heterogeneity precluded biochemical MA

AMSTAR-2 Rating: Moderate confidence

Key Caveats for Interpretation: Contextual PBM framework evidence only; not nanotechnology-specific; results apply to conventional PBMT devices; cannot be extrapolated to Taopatch® without independent characterisation of emission and irradiance

Study 14

First Author, Year, Reference: Vanin, 2018 [Ref. 31 = Ref. 48 in Discussion — see footnote B]

Country: Brazil/International

Domain: PBM — muscular performance

Study Design: Systematic review + meta-analysis

Evidence Stratum: Stratum 4a

Included Studies: 39 RCTs total; 28 included in MA; 861 participants total

Intervention Category: PBMT (LLLT and/or LED; pre-exercise and exercise-concomitant; 655–950 nm range)

Primary Outcome Measures: Time to exhaustion; number of repetitions; isometric peak torque; blood lactate

Key Quantitative Results: ↑ Muscular performance (time to exhaustion, repetitions, torque); ↓ fatigue-related lactate; largest effect: immediate pre-exercise application; 20–60 J (small muscles); 60–300 J (large muscles); quality of evidence: very low to moderate

AMSTAR-2 Rating: Moderate confidence

Key Caveats for Interpretation: Contextual PBM framework evidence; not nanotechnology-specific; low-to-moderate quality of included evidence; extrapolation to Taopatch® requires independent dosimetric confirmation

Study 15

First Author, Year, Reference: Leal-Junior, 2019 [Ref. 30 = Ref. 47 in Discussion]

Country: Brazil/International

Domain: PBM — clinical dosimetry recommendations

Study Design: Systematic review + clinical recommendations synthesis

Evidence Stratum: Stratum 4a

Included Studies: Post-2018 RCTs + prior SR/MA data (n of included primary studies NR in source abstract)

Intervention Category: PBMT (660–905 nm; recommended 20–50 J/muscle group pre-exercise)

Primary Outcome Measures: Exercise performance; post-exercise recovery

Key Quantitative Results: Pre-exercise PBMT at optimal dosimetric parameters (wavelength 660–905 nm; 20–50 J/muscle) = most consistent ergogenic results; dosimetry identified as primary determinant of efficacy; further high-quality RCTs required

AMSTAR-2 Rating: Low confidence (narrative recommendation component; protocol registration status unclear)

Key Caveats for Interpretation: Dosimetric guidance for conventional PBMT; not nanotechnology-specific; dosimetric parameters specifically relevant because Taopatch® irradiance has not been independently measured and cannot be compared to these benchmarks

Study 16

First Author, Year, Reference: Dutra, 2022 [Ref. 28 = Ref. 45 in Discussion]

Country: Brazil/International

Domain: PBM — mode-specific exercise performance

Study Design: Systematic review + meta-analysis

Evidence Stratum: Stratum 4a

Included Studies: 37 individual studies; 78 exercise performance measurements; 586 participants

Intervention Category: PBMT (characterised devices with defined dosimetric parameters)

Primary Outcome Measures: Muscle endurance; cycling time to exhaustion; muscle strength; sprint; running; swimming

Key Quantitative Results: ↑ Muscle endurance: SMD 0.27 (95% CI 0.12–0.41; $p < 0.01$); ↑ Cycling time to exhaustion: SMD 0.35 (95% CI 0.10–0.59; $p < 0.01$); no significant effect on muscle strength, running, swimming performance; mode-specific and parameter-dependent effects

AMSTAR-2 Rating: Moderate confidence

Key Caveats for Interpretation: Contextual PBM framework evidence; effect sizes are mode-specific; swimming and strength show no significant effect; not nanotechnology-specific

Study 17

First Author, Year, Reference: De Marchi, 2022 [Ref. 29 = Ref. 46 in Discussion]

Country: Brazil/International

Domain: PBM — exercise-induced oxidative stress

Study Design: Systematic review + meta-analysis

Evidence Stratum: Stratum 4a

Included Studies: n of included RCTs NR in source abstract; healthy human subjects

Intervention Category: PBMT (characterised devices; pre-exercise and post-exercise)

Primary Outcome Measures: Exercise-induced lipid peroxidation; protein carbonylation; SOD activity; catalase activity

Key Quantitative Results: ↓ Lipid peroxidation; ↓ protein carbonylation; ↑ SOD activity; ↑ catalase activity; PBMT attenuates exercise-induced oxidative stress and enhances endogenous antioxidant enzyme response; specific SMD and 95% CI available in source

AMSTAR-2 Rating: Moderate confidence

Key Caveats for Interpretation: Contextual PBM framework evidence; provides mechanistic plausibility for PBM antioxidant pathway; not nanotechnology-specific

STRATUM 4a — NARRATIVE/COMPREHENSIVE REVIEWS (n = 7)

Study 18

First Author, Year, Reference: Pisanic, 2014 [Ref. 14]

Country: USA

Domain: QD-based biosensing

Study Design: Narrative review

Evidence Stratum: Stratum 4a (narrative subtype)

Review Scope: In vitro and preclinical QD diagnostics and detection (multiple formulations)

Key Topics: Detection sensitivity; photostability; multiplexing

Key Findings: Superior sensitivity, photostability, and multiplexing capacity vs. organic fluorophores; foundational QD biosensing characterisation

Narrative Quality Assessment: Clear review question; broad evidence coverage; selective citation pattern possible; search strategy NR; CoI NR

Key Caveats: No systematic search; not specific to sport applications; foundational technical reference

Study 19

First Author, Year, Reference: Seshadri, 2019 [Ref. 9]

Country: USA

Domain: Wearable nanosensors

Study Design: Narrative review

Evidence Stratum: Stratum 4a (narrative subtype)

Review Scope: Athlete workload monitoring technologies; nano-electrochemical sweat sensors for real-time physiology

Key Topics: Internal/external athlete workload monitoring; sweat lactate, glucose, electrolyte monitoring

Key Findings: Nanomaterial-based electrochemical sensors identified as most promising emerging technology for non-invasive real-time athlete physiological monitoring

Narrative Quality Assessment: Clear question; search partially described; no formal eligibility criteria; CoI NR

Key Caveats: Narrative review; no formal systematic search; AMSTAR-2 not applicable

Study 20

First Author, Year, Reference: Pirzada, 2019 [Ref. 10]

Country: UK/Turkey

Domain: Wearable nanosensors

Study Design: Comprehensive review

Evidence Stratum: Stratum 4a (narrative subtype)

Review Scope: Healthcare and sport biosensing using CNT/graphene-based transducers and biosensor platforms

Key Topics: Detection limit; metabolite selectivity; sport applicability

Key Findings: Orders-of-magnitude lower detection limit vs. conventional electrode materials; high selectivity for exercise-relevant metabolites; direct sport applicability confirmed

Narrative Quality Assessment: Clear question; broad coverage; no systematic search; CoI declared

Key Caveats: Comprehensive but not systematic; extrapolation to in-field athlete monitoring requires further human validation

Study 21

First Author, Year, Reference: Wagner, 2019 [Ref. 13]

Country: USA

Domain: QD-based biosensing

Study Design: Comprehensive review

Evidence Stratum: Stratum 4a (narrative subtype)

Review Scope: Biomedical and preclinical QD applications (multiple formulations: CdSe, CdTe, InP, carbon QDs, GQDs)

Key Topics: Biomedical applicability; in vivo feasibility; cytotoxicity profile

Key Findings: >10,000 publications in QD biomedical applications; CdSe/CdTe cytotoxicity and limited in vivo clearance data = primary clinical translation barriers; heavy-metal-free alternatives in development

Narrative Quality Assessment: Clear question; broad coverage; no systematic search; CoI declared

Key Caveats: Not sport-specific; cytotoxicity barrier critical for any wearable athlete monitoring application involving QDs

Study 22

First Author, Year, Reference: Li MY, 2025 [Ref. 2]

Country: China

Domain: Cross-domain nanotechnology in sport

Study Design: Comprehensive review

Evidence Stratum: Stratum 4a (narrative subtype)

Review Scope: Multiple sport domains (wearable sensors, functional fabrics, sports medicine, equipment engineering)

Key Topics: Wearable sensors; functional fabrics; sports medicine; equipment engineering

Key Findings: Wearable sensors, functional fabrics, and sports medicine identified as most technologically developed application domains for nanotechnology in sport

Narrative Quality Assessment: Clear question; broad domain coverage; no systematic search; CoI declared

Key Caveats: Most directly relevant broad review to this scoping review topic; confirms domain prioritisation consistent with present findings

Study 23

First Author, Year, Reference: Fathy, 2025 [Ref. 37]

Country: USA/International

Domain: Wearable nanosensors

Study Design: Comprehensive review

Evidence Stratum: Stratum 4a (narrative subtype)

Review Scope: Flexible and wearable potentiometric sensor platforms; CNT-based solid-contact ISEs and nanoporous carbon transducers for multi-analyte sweat monitoring

Key Topics: Multi-analyte sweat monitoring (Na^+, K^+, pH, NH_4^+, lactate)

Key Findings: Athlete sweat analysis confirmed as primary application domain; exercise-validated potentiometric CNT systems demonstrated; real-time multi-analyte monitoring feasible in exercise contexts

Narrative Quality Assessment: Clear question; search partially described; broad evidence coverage; CoI NR

Key Caveats: Strong technical foundation; translational validation in athletic competition conditions still limited

Study 24

First Author, Year, Reference: Tiwari, 2021 [Ref. 27]

Country: India

Domain: QD-based biosensing

Study Design: Comprehensive review

Evidence Stratum: Stratum 4a (narrative subtype)

Review Scope: Healthcare and preclinical QD applications (diagnostics, drug delivery, imaging)

Key Topics: Healthcare advancement potential; translational feasibility; cytotoxicity

Key Findings: Wide potential applicability across healthcare and diagnostics; cytotoxicity and regulatory compliance = primary barriers to clinical translation

Narrative Quality Assessment: Clear question; broad coverage; search methods NR; CoI declared

Key Caveats: Not sport-specific; cytotoxicity and regulatory barriers consistently confirmed across multiple QD narrative reviews

STRATUM 4b — MATERIALS SCIENCE STUDIES (n = 4)

Study 25

First Author, Year, Reference: Harifi, 2017 [Ref. 3]

Country: Iran

Domain: CNT-based sports equipment and textiles

Study Design: Narrative review of published materials science evidence

Evidence Stratum: Stratum 4b

Study Model: Lab specimens and published materials data (sports racquet frames, functional textiles)

Outcome Measures: Deformation resistance; frame stiffness; functional textile properties

Key Quantitative Results: ~50% increase in deformation resistance vs. standard carbon racquets; CNT integration improves structural and functional textile properties for sport

Narrative Materials Appraisal: Broad materials coverage; no formal systematic methodology; no independent mechanical testing reported; performance extrapolation to elite athletes inferential

Key Caveats: Technology-enabling outcomes only; direct athlete performance data absent

Study 26

First Author, Year, Reference: Nurazzi, 2021 [Ref. 4]

Country: Malaysia

Domain: CNT-based sports equipment

Study Design: Systematic review (materials science focus)

Evidence Stratum: Stratum 4b

Study Model: Lab specimens (polymer composite test pieces; varied CNT loading fractions)

Outcome Measures: Young's modulus; tensile strength; wear resistance (block-on-ring testing)

Key Quantitative Results: Consistent increase in Young's modulus and tensile strength with increasing CNT content; +50% wear resistance vs. neat polymer matrix; data reproducible across multiple composite configurations

Narrative Materials Appraisal: Quantitative materials data; validated mechanical testing protocols; PICOS not applicable; sport-performance extrapolation inferential

Key Caveats: Technology-enabling outcomes; sport-performance extrapolation not validated in athlete trials; Young's modulus and tensile strength improvements are mechanically plausible preconditions for performance benefit

Study 27

First Author, Year, Reference: Miao, 2023 [Ref. 5]

Country: China

Domain: CNT-based sports equipment

Study Design: Materials science + computational (finite element/computational modelling) study

Evidence Stratum: Stratum 4b

Study Model: Lab specimens and finite element/computational models (optimised CNT/graphene composite configurations)

Outcome Measures: Stiffness-to-weight ratio; energy transfer efficiency; lightweight-strength trade-off index

Key Quantitative Results: Significant optimisation of lightweight-strength trade-off; improved bicycle frame stiffness-to-weight ratio and energy transfer efficiency vs. conventional carbon composites

Narrative Materials Appraisal: Quantitative materials and computational data; limited clinical translation discussion; performance extrapolation to elite athletes is inferential

Key Caveats: Computational model; technology-enabling outcomes only; competitive athlete validation required

Study 28

First Author, Year, Reference: Yamada, 2011 [Ref. 36]

Country: Japan

Domain: Wearable nanosensors

Study Design: Materials science characterisation + proof-of-concept (PoC) human demonstration

Evidence Stratum: Stratum 4b (with limited PoC human component)

Study Model: Lab characterisation + individual human volunteers (PoC demonstration; no formal clinical cohort)

Sample Size (human PoC component): NR as formal clinical cohort; individual demonstrations only

Outcome Measures: Strain range (% elongation at failure); gauge factor; human motion detection fidelity (joint movement, breathing, speech)

Key Quantitative Results: Up to 280% strain range (approximately 50× conventional metal strain gauges); high gauge factor; successfully detected full range of complex human movements, breathing patterns, and speech; landmark nanosensor characterisation study

Narrative Materials Appraisal: Landmark materials science publication; gold-standard SWCNT strain sensor characterisation; human component = PoC only; formal clinical sample size not reported

Key Caveats: Human component is PoC demonstration only; not a clinical study; technological feasibility stage; landmark reference establishing SWCNT sensor performance parameters relevant to athlete motion monitoring

SUPPLEMENTARY MATERIAL S5

Structured Narrative Quality Appraisal Criteria

Applied to Stratum 4a narrative/comprehensive reviews (n = 7) and Stratum 4b materials science studies (n = 4). These criteria are provided in full in accordance with Reviewer 2 Major Comment #11. The frameworks are acknowledged to be less structured and less validated than the RoB 2, NOS, SYRCLE, and AMSTAR-2 tools applied to Strata 1–3 and Stratum 4a true systematic reviews/meta-analyses respectively; this constitutes an explicitly acknowledged limitation of the quality appraisal for these subgroups.

S5.1. Structured Narrative Quality Appraisal Framework: Narrative and Comprehensive Reviews

Applicable to: Stratum 4a narrative/comprehensive reviews (Studies 18–24; n = 7)

Basis: Adapted from guidance on critical appraisal of narrative reviews (Grant MJ, Booth A. A typology of reviews: an analysis of 14 review types and associated methodologies. Health Info Libr J. 2009;26(2):91–108); and from the Scale for the Assessment of Narrative Review Articles (SANRA) (Baethge C et al. SANRA — a scale for the quality assessment of narrative review articles. Res Integr Peer Rev. 2019;4:5).

Criteria (each assessed as Met / Partially Met / Not Met):

Domain	Criterion	Operational Definition	Assessment Options
Domain A	Clarity of the review question	The review explicitly states a focused, answerable question or objective that specifies the population/topic, concept, and context	Met: question fully stated; Partially Met: question implied but not explicitly formulated; Not Met: no discernible review question
Domain B	Description of search methods	The review describes the databases or sources searched, search terms or strategy used, and date of the most recent search	Met: databases, terms, and date fully described; Partially Met: partial description of search process (e.g., databases named but terms or date absent); Not Met: no search methodology described
Domain C	Criteria for inclusion and exclusion of cited evidence	The review specifies explicit criteria for including or excluding studies or sources; or, if such criteria are not pre-specified, the basis for citation selection is described	Met: explicit eligibility criteria stated; Partially Met: selection rationale implied or partially described; Not Met: no inclusion/exclusion rationale provided (narrative selection only)
Domain D	Transparency of synthesis methods	The review describes how cited evidence was interpreted, integrated, or summarized; provides a logical and reproducible synthesis	Met: synthesis process described; Partially Met: synthesis process partially described or qualitative only; Not Met: synthesis methods entirely absent
Domain E	Currency and breadth of cited evidence	The review cites evidence up to a recent date (within 5 years of publication for domains	Met: recent, comprehensive evidence base; Partially Met: predominantly

		with active literature); covers the breadth of evidence types relevant to the topic	current but gaps identifiable; Not Met: predominantly outdated or selectively narrow evidence base
Domain F	Disclosure of conflicts of interest	The review explicitly declares or denies any conflicts of interest for all authors	Met: conflicts of interest disclosed or declared absent; Not Met: no conflict of interest declaration present

Overall rating assignment (for descriptive purposes only; not equivalent to AMSTAR-2 confidence ratings):

- Methodologically transparent: All six criteria Met or predominantly Met (≥4 Met)
- Partial transparency: Mixed (2–3 criteria Met)
- Limited transparency: Predominantly Not Met (≤1 criterion Met)

Results of application to included Stratum 4a narrative/comprehensive reviews:

Study	Domain A (Question)	Domain B (Search)	Domain C (Inclusion criteria)	Domain D (Synthesis)	Domain E (Currency/Breadth)	Domain F (CoI)	Overall Rating
Pisanic 2014 [14]	Met	Not Met	Partially Met	Partially Met	Partially Met (2014; foundational)	Not Met	Partial transparency
Seshadri 2019 [9]	Met	Partially Met	Not Met	Partially Met	Met	Not Met	Partial transparency
Pirzada 2019 [10]	Met	Not Met	Not Met	Partially Met	Met	Met	Partial transparency
Wagner 2019 [13]	Met	Not Met	Not Met	Met	Met	Met	Partial transparency
Li MY 2025 [2]	Met	Not Met	Not Met	Partially Met	Met	Met	Partial transparency

Fathy 2025 [37]	Met	Partially Met	Not Met	Partially Met	Met	Not Met	Partial transparency
Tiwari 2021 [27]	Met	Not Met	Not Met	Partially Met	Met	Met	Partial transparency

Interpretation note: All seven narrative/comprehensive reviews were classified as "Partial transparency." This is consistent with the inherent methodological limitations of narrative and comprehensive reviews relative to true systematic reviews. The primary limiting factors across all seven were: absence of fully described systematic search strategies (Domain B); absence of explicit pre-specified eligibility criteria (Domain C); and incomplete conflict of interest disclosure in some studies (Domain F). These limitations are acknowledged in the main manuscript (Section 2.6 and Section 4.5). Findings from these seven studies are incorporated as contextual framework evidence only (Stratum 4a), and are explicitly distinguished from primary direct evidence (Strata 1–2) throughout the synthesis.

S5.2. Narrative Quality Appraisal Framework: Materials Science Studies

Applicable to: Stratum 4b materials science studies (Studies 25–28; n = 4)

Basis: Adapted from guidance on quality reporting in materials science studies (reporting standards for mechanical testing: ISO 6892-1 for tensile testing; ASTM E8/E8M; ASTM G65 for wear resistance); and from materials characterization completeness criteria consistent with best-practice reporting in nanomaterials science literature.

Criteria (each assessed as Adequate / Partial / Inadequate):

Domain	Criterion	Operational Definition	Assessment Options
Domain I	Completeness of nanomaterial characterization (physicochemical)	The study provides characterization of the nanomaterial using at least two independent methods from: electron microscopy (SEM/TEM), Raman spectroscopy, X-ray diffraction (XRD), Fourier-transform infrared spectroscopy (FTIR), thermogravimetric	Adequate: ≥2 independent characterization methods used; Partial: 1 method described; Inadequate: nanomaterial characterization absent or referenced only to prior work

		analysis (TGA), or equivalent	
Domain II	Completeness of mechanical and functional outcome reporting	The study reports quantitative mechanical outcomes (e.g., Young's modulus, tensile strength, wear resistance, gauge factor, stiffness-to-weight ratio) with appropriate statistical descriptors (mean, SD or variance, and range)	Adequate: quantitative outcomes with full statistical descriptors; Partial: quantitative outcomes with partial statistical descriptors; Inadequate: qualitative description only or reference to figures without tabulated data
Domain III	Reproducibility of testing conditions	The study describes testing conditions (specimen preparation, loading rates, temperature, humidity, cycling protocols) in sufficient detail to allow independent replication	Adequate: full testing condition description enabling replication; Partial: partial description of testing conditions; Inadequate: testing conditions insufficiently described for replication
Domain IV	Relevance and specificity of outcomes to athletic performance	The study outcomes (material properties, sensor performance metrics, computational model outputs) have a clear, directly articulable relationship to athletic performance applications through a plausible mechanistic pathway	Adequate: direct and explicitly described mechanistic link to athletic performance; Partial: plausible but incompletely articulated link; Inadequate: no clear relationship to athletic performance articulated
Domain V	Transparency of reporting (figures, raw data, and statistical analysis)	The study provides sufficient raw data, figures with axis labels and error bars, and statistical analysis details to allow assessment of quantitative claims	Adequate: full data transparency; Partial: partial data transparency; Inadequate: data presentation insufficient for

			independent assessment
Domain VI	Absence of unsupported extrapolation to clinical or competitive athletic populations	The study does not overstate the implications of materials science findings as demonstrated performance benefits in elite athletes; inferential steps from material properties to athletic performance are appropriately qualified	Adequate: performance implications appropriately qualified as inferential; Partial: some unsupported extrapolation present but acknowledged; Inadequate: material property findings presented as demonstrated performance benefits without appropriate qualification

Overall rating assignment (for descriptive purposes only):

- High methodological rigour: ≥4 criteria Adequate; relevant to sport context
- Moderate methodological rigour: 2–3 criteria Adequate; some limitation
- Limited methodological rigour: ≤1 criterion Adequate

Results of application to included Stratum 4b materials science studies:

Study	Domain I (Characterization)	Domain II (Quantitative outcomes)	Domain III (Reproducibility)	Domain IV (Sport relevance)	Domain V (Data transparency)	Domain VI (No unsupported extrapolation)	Overall Rating
Harifi 2017 [3]	Partial (referenced to prior work)	Partial	Inadequate	Adequate	Partial	Partial	Limited-to-Moderate
Nurazzi 2021 [4]	Adequate	Adequate	Adequate	Adequate	Adequate	Adequate	High

Miao 2023 [5]	Partial	Adequate	Partial	Adequate	Adequate	Partial	Moderate
Yamada 2011 [36]	Adequate	Adequate	Adequate	Adequate	Adequate	Adequate	High

Interpretation note: Nurazzi 2021 and Yamada 2011 achieved the highest methodological rigour ratings, reflecting validated mechanical testing protocols (Nurazzi) and comprehensive sensor characterisation with transparent reporting (Yamada). Harifi 2017 received a limited-to-moderate rating due to the absence of independent mechanical testing and reliance on referenced prior characterisation data. Miao 2023 received a moderate rating with partial limitations in nanomaterial characterisation completeness and extrapolation qualification. For all four studies, the key translational limitation is the absence of controlled athlete performance trials; technology-enabling outcomes (material properties, sensor metrics) cannot be equated with demonstrated performance benefits. This is explicitly stated throughout the main manuscript synthesis.
